# Dysphagia After Total Laryngectomy: An Exploratory Study and Clinical Phase II Rehabilitation Trial with the Novel Swallowing Exercise Aid (SEA 2.0)

**DOI:** 10.1007/s00455-024-10673-7

**Published:** 2024-04-01

**Authors:** Marise Neijman, Frans Hilgers, Michiel van den Brekel, Rob van Son, Martijn Stuiver, Lisette van der Molen

**Affiliations:** 1 Department of Head and Neck Oncology and Surgery, The Netherlands Cancer Institute, Plesmanlaan 121, 1066 CX Amsterdam, The Netherlands; 2 Center for Quality of Life and Division of Psychosocial Research and Epidemiology, The Netherlands Cancer Institute, Plesmanlaan 121, 1066 CX Amsterdam, The Netherlands; 3 Amsterdam Center for Language and Communication (ACLC), University of Amsterdam, Binnengasthuisstraat 9, 1012 ZA Amsterdam, The Netherlands; 4 Department of Oral and Maxillofacial Surgery, Amsterdam University Medical Center, Meibergdreef 9, 1105 AZ Amsterdam, The Netherlands

**Keywords:** Head and neck cancer, Total laryngectomy, Dysphagia, Muscle strength exercises, Swallowing exercise aid, Rehabilitation

## Abstract

The aims of this exploratory study and clinical phase II trial were to assess the specific nature and extent of dysphagia in laryngectomized patients with self-reported dysphagia, and its rehabilitation potential using the novel Swallowing Exercise Aid (SEA 2.0). Twenty laryngectomized patients participated in a six-week exercise program with the SEA 2.0. Exercises consisted of Chin Tuck Against Resistance (CTAR), Jaw Opening Against Resistance (JOAR), and Effortful Swallow Against Resistance, conducted three times per day. Swallowing was assessed at baseline (T0), six (T1) and 14 (T2) weeks, consisting of patient-reported outcomes, BMI, videofluoroscopy, swallowing capacity, and muscle strength measurements. Dysphagia significantly impacts quality of life, with impaired swallowing speed and bolus propulsion as main reported issues. Subjective dysphagia parameters, swallowing capacity, and pharyngeal residue clearance were reduced, but BMI was normal. Muscle and tongue strength were within normal ranges. All participants managed to use the SEA 2.0. Adherence was 95%. At T1, subjective swallowing parameters (MDADI and EAT-10) showed clinically relevant improvements. Objectively, CTAR and JOAR strength increased with 27.4 and 20.1 Newton, respectively. Also, swallowing capacity (from 2.4 g/s to 3.8 g/s) and pharyngeal residue clearance improved. At T2, results were slightly lower than at T1, but still better than at baseline. Dysphagia in laryngectomized patients affects quality of life, but swallowing can be improved with a six-week rehabilitation program using the novel SEA 2.0. Adherence was excellent and several subjective and objective swallowing parameters improved.

**Supplementary Information:**

The online version contains supplementary material available at 10.1007/s00455-024-10673-7.

## Introduction

Presently, indications for total laryngectomy (TL) are primary treatment for advanced laryngeal or hypopharyngeal cancer, salvage surgery for recurrence, or severe functional or aspiration problems after organ-preservation treatment [1, 2].

Standard TL procedure includes removing the entire larynx, hyoid bone, and infrahyoid muscles. The upper and lower airways are disconnected, resulting in a tracheostoma in the neck. The transected suprahyoid muscles are mostly reattached to the superior constrictor pharyngeus muscles, and often, the cricopharyngeal muscles are myotomized. If possible, a new pharynx (neopharynx) is created by primary mucosal (T/Y shape, horizontal or vertical) closure, with or without closing the constrictor muscles in the midline, or by means of a flap reconstruction [3].

After surgery, the patient has to adapt to the altered anatomy and its lifelong consequences, leading to physical, emotional, psychological, and social changes and affecting average daily functioning and quality of life [4–8]. The most obvious consequence is that patients lose their natural voice source and have to learn speaking with a substitute voice such as an electrolarynx, esophageal, or tracheoesophageal voice. Acoustically and perceptually tracheoesophageal speech is favorable and presently the method of choice [9]. Two less obvious consequences are the loss of upper airway function (moistening, heating, and filtering of air), resulting in pulmonary problems and loss of olfaction. Fortunately, these functions can be rehabilitated with heat and moisture exchangers and the Nasal Airflow Inducing Maneuver (NAIM), respectively [10, 11].

Another impacting consequence is the changed swallowing physiology and biomechanics. Although patients expect and adapt to some diminution in levels of swallowing functioning after such significant surgery, Maclean et al. found long-term self-reported swallowing problems in 72% of patients after TL [12]. Patients reported complaints about food sticking in the throat, regurgitation, increased swallow attempts, tightness, and prolonged mealtimes [13, 14]. These complaints can be attributed to the altered anatomy and physiology of the neopharynx. After TL, reduced peak mid-pharyngeal pressures, lower hypopharyngeal peak (contractile) pressures, and increased hypopharyngeal bolus pressures in the neopharynx are observed analyzing the swallowing function with videofluoroscopy in combination with high-resolution manometry [15]. Furthermore, velopharyngeal insufficiency, pseudodiverticulum/pseudoepiglottis, stricture, high incidence of pharyngeal reflux, and abnormalities in peristalsis can negatively influence the swallowing function [14, 16, 17]. Maclean et al. also found a significantly reduced pharyngeal diameter at the fluoroscopy of 7.2 mm in sagittal and 7.5 mm in AP projections in the laryngectomized participants versus 10.6 mm in sagittal and 15.7 mm in AP projections in the aged controls [15, 18]. According to Maclean et al. and Harris et al., surgical closure techniques might influence swallowing outcomes [15, 19]. For instance, preserving as much mucosa as possible and using a primary closure technique may positively influence pharyngeal transit times and swallowing function [19]. Besides, mucosa-and-muscle closure appears to result in superior swallowing function compared to mucosa-alone closure [15]. The altered transit of the bolus through the pharynx, stenosis of the neopharynx, insufficient pressure build-up at the tongue base, loss of coordinated muscular contraction in the neopharynx, and an increased pharyngeal resistance secondary to the altered anatomy affect the swallowing function too [20].

The question arises whether dysphagia after TL can be rehabilitated, beyond the not seldom required dilatation of strictures, e.g., in 22.8% of the patients according to a recent study in the Netherlands [21]. Classically, dysphagia rehabilitation aims to maintain or improve adequate oral intake and to ensure airway protection, the latter obviously no longer being an issue after TL. Different non-surgical techniques to rehabilitate dysphagia include dietary modification, compensatory techniques, (strengthening) exercises for (swallowing) muscles, and medical treatment/dilatation. The Shaker exercise, Masako maneuver, and Effortful swallow are swallowing maneuvers or exercises commonly used in dysphagia rehabilitation [22–27]. These exercises target specific aspects of the swallowing process, and all aim at improving swallowing function by enhancing swallowing safety, bolus propulsion, swallow muscle strength, and overall swallowing efficiency [22–25, 28, 29]. An MRI study by Karsten et al. investigated the muscles activated by conventional swallowing exercises in healthy adults. For the Shaker exercise, they could demonstrate a significant activation of the suprahyoid, infrahyoid, and sternocleidomastoid muscles [30]. For the conventional Effortful Swallow exercise and the Masako maneuver, they did not find such activation. In laryngectomized patients, the infrahyoid muscles have been removed together with the larynx and the suprahyoid muscles are sutured to the constrictor pharyngeal muscles around the neopharynx. However, it is not known whether the suprahyoid musculature still has an (elevating/widening) effect on the reattached neopharynx and whether these muscles are still trainable, e.g., with the Shaker exercise, for achieving more efficient swallowing.

If increasing muscle strength is of potential value for improving swallowing, the rehabilitation exercises should meet the following criteria: the involved musculature should play a role in propulsion and clearance, and there should be an overload, i.e., the musculature should be targeted specifically. Also, to ensure an effective increase in muscle strength, resistance should progress as strength improves. Conventional exercises are done without external load, and consequently progressive resistance cannot be applied.

The Swallow Exercise Aid (SEA) was trialed for the first time in 2015. The SEA device was at this time constructed with commercially available parts, i.e., the TheraBite Jaw Mobilization device complemented with a TheraBite ActiveBand made out of silicone rubber (Atos Medical, Horby, Sweden) [31]. This handheld device allows increasing resistances from 10 to 50 Newton (one band) and up to 100 Newton with two bands and enables three swallow muscle-strengthening exercises, i.e., the Chin Tuck Against Resistance (CTAR), the Jaw Opening Against Resistance (JOAR), and the Effortful Swallow Against Resistance (ESAR). The CTAR exercise is executed by pressing the chin downward against the chin bar. Karsten et al. found during the MRI assessment mentioned above that the CTAR exercise activated the suprahyoid muscles, infrahyoid muscles, and sternocleidomastoid muscle [30]. The JOAR exercise is performed by pressing the mandible down while opening the mouth to compress the chin bar onto the chest bar. Karsten et al. found the JOAR exercise activating the lateral pterygoid muscles and suprahyoid muscles [30]. The ESAR is performed with pressing the mouth open at 50% of the maximum while keeping the lips closed, hold the mouth in position against the resistance of the chin bar and then swallow. Karsten et al. found that the ESAR exercise activates the lateral pterygoid muscle, suprahyoid muscles, and infrahyoid muscles [30].

Training with the SEA meets two principles of strength training, i.e., it is specifically recruiting various muscles involved in swallowing and provides adjustable resistance. An additional benefit is that it provides direct feedback to the patients by an audible and tactile click when the movement is completed. The aforementioned MRI study showed that training with the SEA, just as the Shaker exercise, activated the suprahyoid muscles, infrahyoid muscles, and sternocleidomastoid muscles, but additionally activated the lateral pterygoid musculature [30].

Kraaijenga et al. evaluated the effectiveness and feasibility of a 6-week/3 times daily training program with the SEA in healthy senior participants [31]. After the training period, they found high compliance (83%) and a significant increase in Chin Tuck, Jaw Opening, and (IOPI) tongue strengths. Furthermore, the MRI assessment showed an increased median muscle volume of the mylohyoid, geniohyoid, and anterior belly of the digastric muscles combined [31]. In 2016, the same training protocol was used in long-term head and neck cancer survivors who suffered from chronic dysphagia. After the six-week training period, high compliance (97%) and feasibility (88%) were found. Furthermore, objective and subjective effects of progressive load on muscle strength and swallowing function in these long-term therapy refractory dysphagia sufferers were found in 85% of the patients [32].

However, the first-generation SEA device used in previous studies is considered not to be very suitable for patients with a tracheostoma because it could cover that and make breathing difficult during exercising. Another disadvantage of the first-generation SEA is that, although it is possible to vary the resistance with the silicon band(s), the position of the bands and thus the training resistances are not well defined. Therefore, the SEA principles have been incorporated into a novel dedicated swallowing rehabilitation device. This device has been developed in collaboration with Atos Medical into a CE-marked, handheld device called the Swallow Exercise Aid 2.0 (SEA 2.0).

To assess dysphagia in TL patients and whether it is possible to rehabilitate this problem, we carried out an exploratory and intervention study in a TL patient population with self-reported dysphagia. The aims of this study were to assess the specific nature and extent of dysphagia in this group of patients, the usability of the novel SEA 2.0 device as a rehabilitation tool, and, in a six-week clinical phase II trial, the feasibility and adherence of the training program and the early and longer-term (eight weeks later) effects on swallowing function.

## Methods

The study was performed at a tertiary Head and Neck Oncology Department and approved by the local Medical Ethical Committee (METC21.0904/N21STL). The guidelines of the Helsinki Declaration were followed, and written informed consent was obtained from each participant before inclusion.

### Participants

Between April 2022 to February 2023, individuals (≥ 18 years) who underwent TL and were experiencing dysphagia were recruited from the own institute and via a notice on the Dutch Patient Association for Head and Neck platform. Participants had to be at least six months post-surgery before enrollment and, if applicable, had completed their postoperative (chemo-) radiotherapy at least six months ago. Pharynx reconstruction method was not a selection criterion. We aimed to include 20 participants based on previous results demonstrated in the healthy senior subjects (Cohen’s *d* > 0.6) [31]. At the end of the enrollment period, 21 participants [17 men (81%), four women (19%)] were included and signed informed consent. One participant (S13) changed his mind after a few days and withdrew from the study before starting, leaving 20 participants for inclusion.

The median age at baseline was 71 years (range 45–78), and the median time after TL was 47 months (range 9–274). Six participants (30%) had a history of stenosis and had undergone one or more dilatations. Nineteen participants were able to maintain an adequate oral diet, and one participant was feeding tube-dependent. Participant characteristics are displayed in Table 1.

**Table 1:** Participant characteristics

Participant			Tumor		Treatment					
	Sex	Age	Location	TNM	Indication	Months since TL	Pharynx closure	Myotomy	Stenosis (dilatations)	Timing (C)RT
S01	M	78	Hypopharynx	T1N1	Functional	47	T/Y shape	No	No (0)	Pre-surgery
S02	M	65	Hypopharynx	pT4aN0	Curative	73	PM	Yes	Yes (2)	Post-surgery
S03	M	75	Larynx	cT2N0	Salvage	75	T/Y shape	Yes	Yes (1)	Pre-surgery
S04	M	73	Hypopharynx	cT3N2b	Curative	40	PM	No	Yes (1)	Pre-surgery
S05	F	62	Trachea	pT4bN0	Curative	35	Horizontal	Yes	No (0)	Post-surgery
S06	M	53	Larynx	pT4N2a	Curative	12	T/Y shape	Yes	No (0)	No
S07	M	72	Larynx	T1bN0	Salvage	94	Vertical	Yes	No (0)	Pre-surgery
S08	M	71	Larynx	cT2N0	Salvage	44	PM	Yes	No (0)	Pre-surgery
S09	M	76	Hypopharynx	T3N2c	Curative	24	ALT	No	Yes (2)	Post-surgery
S10	M	61	Larynx	cT4aN0	Curative	47	T/Y shape	Yes	No (0)	No
S11	M	67	Larynx	cT4aN0	Curative	65	T/Y shape	Yes	No (0)	No
S12	M	77	Larynx	T4N0	Salvage	274	T/Y shape	Yes	No (0)	Pre-surgery
S14	F	45	Larynx	cT3N0	Salvage	22	T/Y shape	Yes	Yes (2)	Pre-surgery
S15	M	50	Hypopharynx	pT4N3b	Curative	33	SCAIF	No	No (0)	Post-surgery
S16	M	66	Larynx	pT4aN0	Curative	16	T/Y shape	Yes	No (0)	Post-surgery
S17	M	63	Larynx	T2N0	Salvage	67	Vertical	Yes	No (0)	No
S18	M	70	Larynx	rT2N0	Salvage	9	T/Y shape	No	No (0)	Post-surgery
S19	F	73	Larynx	T3N2b	Curative	147	T/Y shape	Yes	Yes (2)	No
S20	M	77	Larynx	T4aN0	Salvage	138	Vertical	Yes	No (0)	Post-surgery
S21	F	73	Hypopharynx	T4aN0	Salvage	87	Gastric Pull-up	No	No (0)	No
Median (Range)		71 (45–78)				47 (9–274)				

*TNM* a classifying system for malignancy consisting of T (tumor) N (node) and M (metastasis), *PM* Pectoralis Major Flap, *ALT* Anterolateral Thigh Flap, *SCAIF* Supraclavicular Artery Island Flap, *(C)RT* (Chemo) Radiation Therapy. (S13 withdrew from study before start)

### Multidimensional Assessment Program

A multidimensional assessment program was set up to assess the specific nature and extent of the dysphagia and to investigate whether training with the SEA 2.0 improves subjective and/or objective swallowing function. Different questionnaires/patient-reported outcome measurements (PROMs) and objective assessments were combined. Since none of the measurements, except the Swallowing Outcome After Laryngectomy (SOAL) questionnaire, are developed for TL patients, tools frequently used in the Head and Neck patient population were selected. The PROMs were completed by the participants individually, and the clinician checked if all questions were answered to ensure all data were present. All objective assessments were video-recorded with the SONY ZV-E10 camera placed on a ROLLEI mini M1 tripod. The average time to complete the total assessment was approximately 90 min. All outcome parameters were assessed prior to participation (at baseline, T0), two days after the 6-week training period (T1) and after eight weeks of rest (longer term results, T2).

### Patient-Reported Outcomes

#### Laryngectomy Dysphagia Complaints Inventory

The assessment was started with a study-specific structured interview was held with each participant. The participants were asked about eating, drinking, swallowing, (type of) oral intake, adaptions, duration, anxiety, regurgitation, stenosis, and sensitivity. During the interview, the clinician scored the topics dichotomous as “problem or coping strategy present” or “problem or coping strategy absent.” With the dichotomous outcomes of the interview, a Laryngectomy Dysphagia Complaints Inventory (LDCI) was created (see Table 2).

**Table 2:**
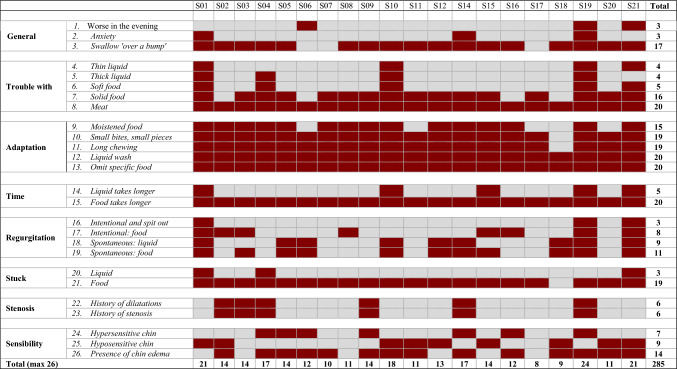
The Laryngectomy Dysphagia complaints inventory—baseline assessment

*Dark red* means that the complaint is present, *gray* means that the complaint is absent

#### Subjective Perspective on General Health

Participants’ health state was assessed with the EQ-5D-5L questionnaire using health profiles. This validated questionnaire focuses on mobility, self-care, usual activities, pain/discomfort, and anxiety/depression and includes five levels (no, slight, moderate, severe, and extreme problems) for each modality. The outcome ranges from − 0.446 to 1.000, with a higher score indicating better health state. The reference score is 0.839 [33]. It also includes a vertical visual analog scale (VAS) ranging from 0 to 100 to record the participants’ self-rated health status.

#### Subjective Perspective on Swallowing Function and Oral Intake

The Functional Oral Intake Scale (FOIS) and International Dysphagia Diet Standardization Initiative (IDDSI) assessed oral intake status and type of diet [34–36]. The FOIS is a reliable and validated tool that ranges from 1 to 7, with nothing by mouth (1) and no oral restriction (7) [34]. The type of diet was classified with the IDDSI and ranges from 0 to 7, with thin liquid (0) and normal solid or easy to chew consistencies (7) [36].

The MD Anderson Dysphagia Inventory (MDADI) questionnaire, validated for Dutch, was used to evaluate the impact of dysphagia on the quality of life. The MDADI consists of twenty statements with five answer options [Strongly Agree (1), Agree (2), No Opinion (3), Disagree (4), Strongly Disagree (5)]. Total scores range from 20 (extremely low functioning) to 100 (high functioning) [37, 38]. This questionnaire is not specifically designed for TL patients and therefore it also includes questions about aspiration that are not relevant for TL patients; however, the TL patients were able to answer those questions with “Disagree” or “Strongly Disagree.”

The Dutch validated Eating Assessment Tool (EAT-10) questionnaire is a self-administered, symptom-specific outcome instrument for dysphagia [39]. It includes ten questions that assess the initial dysphagia severity with five answer options per question [No Problems (0) to Severe Problems(4)]. A score of three or more is considered abnormal [40]. Also this questionnaire is not specifically designed for TL patients since it includes questions about aspiration; however, the TL patients were able to answer those questions with “No Problems.”

The Swallowing Outcomes after Laryngectomy (SOAL) questionnaire focuses on swallowing problems, specifically for laryngectomized patients. The English version of the SOAL has been validated [41], and the Dutch version is currently being tested for validity. The SOAL consists of 17 questions that assess issues patients may experience with their swallowing function after TL, and every question has three answer options [No (0), A Little (1), A Lot (2)]. Lower scores indicate fewer problems and better self-reported overall swallowing function [41].

### Objective Measurements

#### Body Weight, Body Length, and BMI

Body weight and body length were provided by the patients, and the Body Mass Index (BMI) was calculated. The BMI was interpreted as follows: < 18.5 Underweight, ≥ 18.5 and < 25.0 Normal weight, ≥ 25.0 and < 30.0 Overweight, and ≥ 30.0 Obese [42, 43]. Reference values for the distribution of the BMI in seniors (65 + years) in the Netherlands (2022) are: 43.8% Under- and Normal weight, 40% Overweight, and 16.2% Obese [42].

#### Swallowing Assessment

Swallowing function was assessed using two methods. First, videofluoroscopy (VFSS) was used to assess the different phases of the swallow and the amount of residue. All participants were instructed to sit in upright position and to swallow different consistencies in varying amounts of Omnipaque (350 mg I/ml). The protocol included 2 × 10 cc and 1 × 40 cc of thin liquid (IDDSI 0), 2 × 10 cc of extremely thick liquid (IDDSI 4), and 1× (Omnipaque coated) cracker (IDDSI 7) in random order, to avoid a learning effect. The videofluoroscopy videos were recorded at 25 frames per second and exported as AVI file.

For analysis, the Dynamic Imaging Grade of Swallowing Toxicity (DIGEST) was used [44, 45]. The DIGEST is a reliable, validated ordinal scoring tool for pharyngeal dysphagia. The DIGEST uses a Safety and Efficiency Grade to quantify pharyngeal bolus transit. Since laryngectomized participants have no penetration or aspiration issues (unless there is a leaking voice prosthesis), the Safety Grade is scored 0 (Normal; Material does not enter the airway). To assess the Efficiency Grade, first the percentage of pharyngeal residue remaining in the entirety of the pharynx after the initial swallow of each bolus have to be scored (< 10%, 10%–49%, 50%–90%, and > 90%). Then the estimated percentage residue can be converted into the Efficiency Grade (scored from 0 = Normal to 4 = Profound Impaired). Since all participants had a voice prosthesis, the landmarks for the (neo) pharynx were considered from base of the skull up to the vertebra where the voice prosthesis is located. The overall DIGEST Grade is based on the interaction between the Safety Grade (scored as 0 in our participants) and the Efficiency Grade (scored from 0 to 4) and can be interpreted as the severity of dysphagia (0 = Safe and Efficient, 1 = Safe and Mildly Inefficient, 2 = Safe and Moderately Inefficient, 3 = Safe and Severely Inefficient, 4 = Safe and Profoundly Inefficient). A lower score on the DIGEST was interpreted as better swallowing efficiency and reduced residue in the neopharynx. Besides the DIGEST, visual perceptual outcome variables including the presence of a pseudovallecula or pseudoepiglottis, stenosis, regurgitation (nasal, oral, or pharyngeal), pre-swallow posterior spill, tongue base contact against the posterior neopharynx wall, piecemeal deglutition, oral residue, and liquid wash were scored.

The second objective method was the Swallowing Proficiency for Eating and Drinking (SPEAD) test, which measures the swallowing capacity [46]. The SPEAD test is reliable, feasible, and valid to objectify the transport capacity of the upper digestive tract (in grams per second) and has been developed to evaluate and monitor the swallowing capacity in head and neck cancer patients. The SPEAD test contains three subtasks covering the normal range of food consistencies, including thin liquid, thick liquid, and solid texture. The participant was instructed to sit straight in a chair at the table and asked to swallow three food consistencies as quickly and comfortably as possible, with at least 60 s of rest between the different consistencies. The observer kept track of the time and videotaped the participant during the SPEAD test. The videotape was analyzed on total duration (time between substance touching lips until the end of the last swallow), grams swallowed, number of swallows, and number of chews. Different outcomes were calculated: (1) speed of ingestion per consistency (g/s), (2) average swallow volume (g/swallow), and (3) the SPEAD rate (g/s), combining the mean ingestion speed of the three consistencies. Karsten et al. found ‘normal’ SPEAD rate values of 6 g/s (range 2–11 g/s) for healthy participants and 2 g/s (range 0–10 g/s) for Head and Neck cancer patients diagnosed with dysphagia [46].

To ensure blinded analysis, the order of all videofluoroscopy and SPEAD test recordings at baseline, T1, and T2 measurements was randomized by giving each video a random number between 1 and 60. Both key documents were secured with passwords and saved in an independent folder. A pre-analysis consensus meeting between two Speech and Language Pathologists (SLPs) was held. After the consensus meeting, one trained SLP (MN) analyzed all randomized VFSS and SPEAD test video recordings independently. A second SLP (LvdM) scored 10% of the VFSS recordings, and a third SLP (MLA) scored 10% of the SPEAD test video recordings to assess interrater reliability. After three weeks, the first SLP (MN) again scored 10% of the VFSS and SPEAD videos to assess intra-rater reliability.

#### Mouth Opening Assessment

The clinician measured the Maximum Interincisor Opening (MIO) in millimeters using the TheraBite Range of Motion (ROM) scale (Atos Medical, Hörby, Sweden). A mouth opening of ≤ 35 mm was considered trismus [47–49].

#### Tongue and Strength Assessment

The Iowa Oral Performance Instrument (IOPI) was used to measure tongue strength [50]. With IOPI, measuring the maximum tongue pressures (at anterior and posterior locations) and endurance is possible using a small air-filled bulb. Participants were instructed to sit up straight and press the tongue upwards on the air-filled bulb to squeeze the bulb against the hard palate. The IOPI digitally measures pressures in kilopascal (kPa). The normal values of the tongue elevation strength (P-max) fall in about 40–80 kPa with an average of about 63 kPa [51]. After one familiarization session, three maximum tongue pressure trials are obtained for each participant, with approximately 2-min rest period between the tests. The mean maximum pressure of the highest two of three values was calculated and used as the participants’ maximal (anterior) tongue strength. To ensure the exact positioning of the bulb in the mouth, a small rubber band was attached around the silicone tube at the level of the lips.

CTAR and JOAR strengths were measured in Newton with a digital dynamometer (MicroFET™, Biometrics, Almere, the Netherlands), mounted in an adapted ophthalmic examination frame, used to avoid head and chin position variations and ensure consistent measurement, as previously described by Kraaijenga et al. [31, 32]. A superior fixed belt stabilized the participants’ heads, and the height of both the chin rest and the superior belt were adjustable per participant. Participants were instructed to sit upright and press their chin down on the dynamometer as effortful as possible, once with their mouth and teeth closed (like the CTAR) and once by opening their jaw/mouth (like the JOAR). The dynamometer measured the maximal isometric chin tuck and jaw opening strength (in Newton). Both measurements were preceded by one familiarization session to exclude learning curve effects and improve the reliability of the values obtained. After the familiarization session, both measures are repeated three times, with a 60-s rest period between the trials. The highest value of three was considered as the 1 Repetition Maximum (1RM). The 1RM is the maximum amount of force that can be generated in one maximal contraction [52]. The mean pressure of the highest two values was calculated and considered as the participants’ mean strength of both exercises [50].

### The Swallow Exercise Aid (SEA) 2.0

**Fig. 1:**
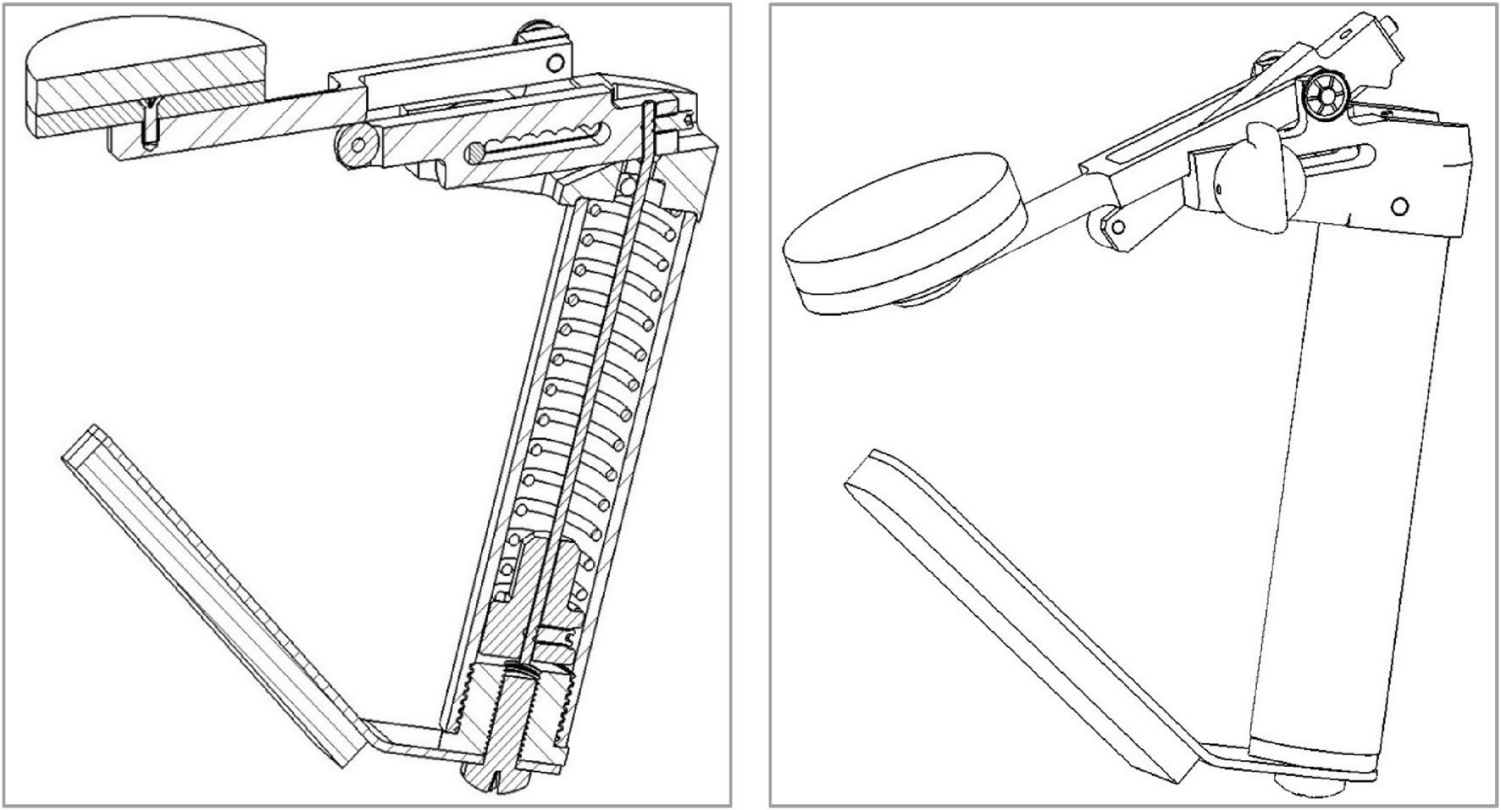
schematically drawing of the mechanism (left) and design (right)of the Swallow Exercise Aid (SEA) 2.0

**Fig. 2:**
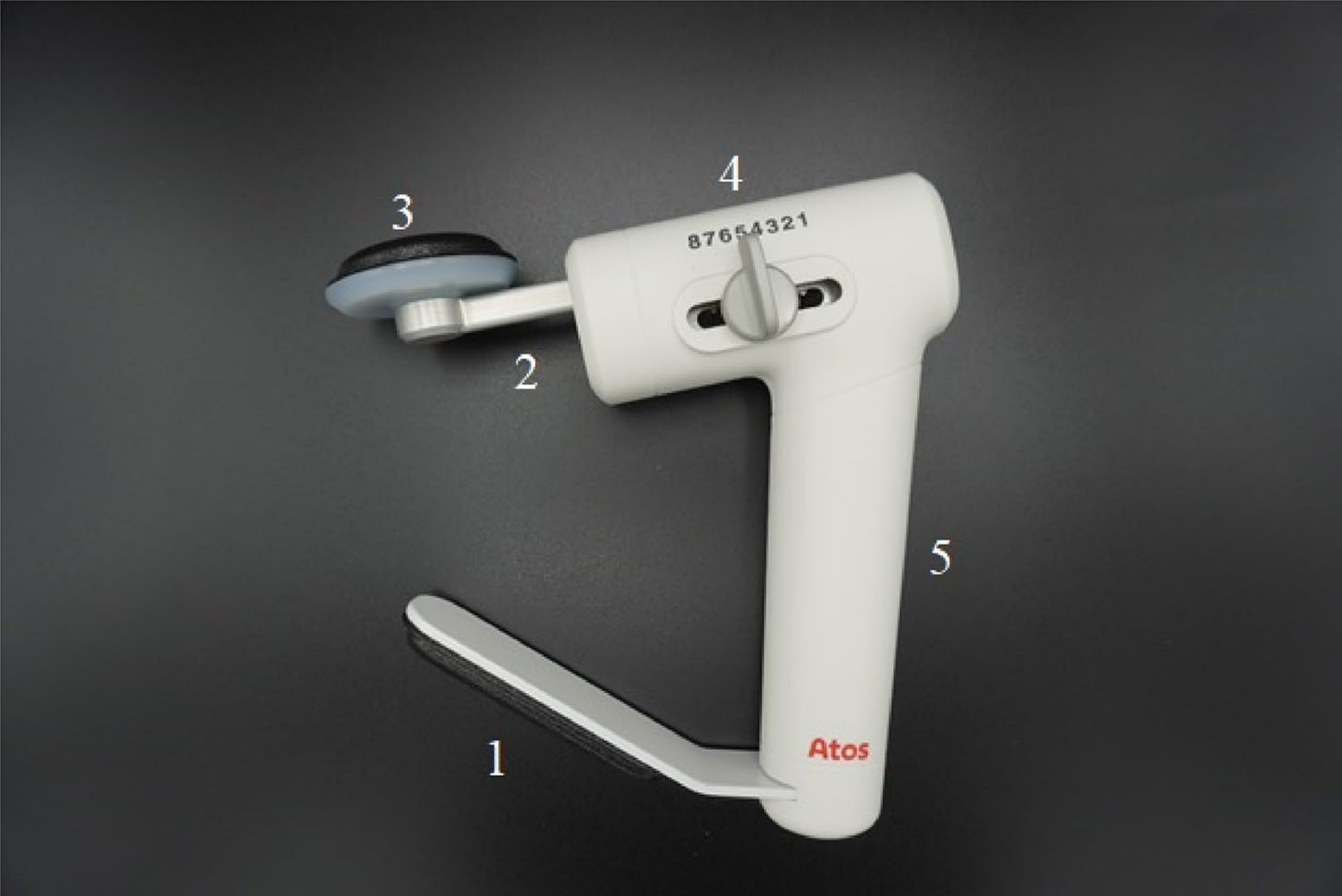
Picture of the novel Swallow Exercise Aid (SEA 2.0). The SEA 2.0 consists of the Chest Bar with Chest Pad (1), Chin Bar (2), Chin Pad (3), Resistance adjustment knob (4), and Handle (5)

**Fig. 3:**
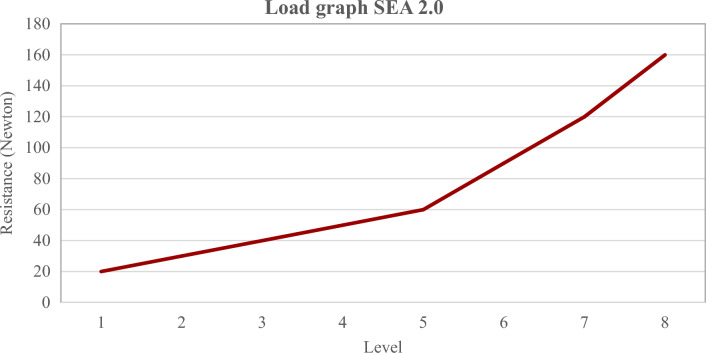
Load graph of the resistance (in Newton) of the novel Swallow Exercise Aid (SEA 2.0) based on 100 pieces measured at ATOS Medical (Hörby, Sweden)

The previously extensively described SEA is further developed into the new SEA 2.0 (see Fig. 1) [31, 32]. The SEA 2.0 consists of the Chest Bar with Chest Pad (1), Chin Bar (2), Chin Pad (3), Resistance adjustment knob (4), and Handle (5) (see Fig. 2). The range of motion of the Chin Bar is 30 mm in total, resulting in enough space for the tracheostoma and breathing. The SEA 2.0 device provides quantifiable adjustable resistance training for swallowing and jaw exercises, and audible and tactile feedback. Unique to this SEA 2.0 is the mechanism that allows a stepwise resistance force in Newton raising from level 1 (20 Newton) to level 8 (150 Newton), which was determined on 100 devices by Atos Medical (Hörby, Sweden) (see Fig. 3). This mechanism makes it possible to adapt the resistance to participants’ capacity and/or performance.

Due to the altered anatomy and the lack of knowledge about how the remaining and reattached muscles would react to the exercises, it was decided that the exercises should be performed at a level that was strenuous but well tolerated according to patients. To obtain the training load, the initial resistance was set at 60–75%1RM as measured with the MicroFET and was then adjusted to achieve a load that was rated as at least strenuous at 30 repetitions in a first practice round. The final starting resistance in Newton was then deducted from the Load graph.

### Exercise Protocol and Logbook

**Fig. 4:**
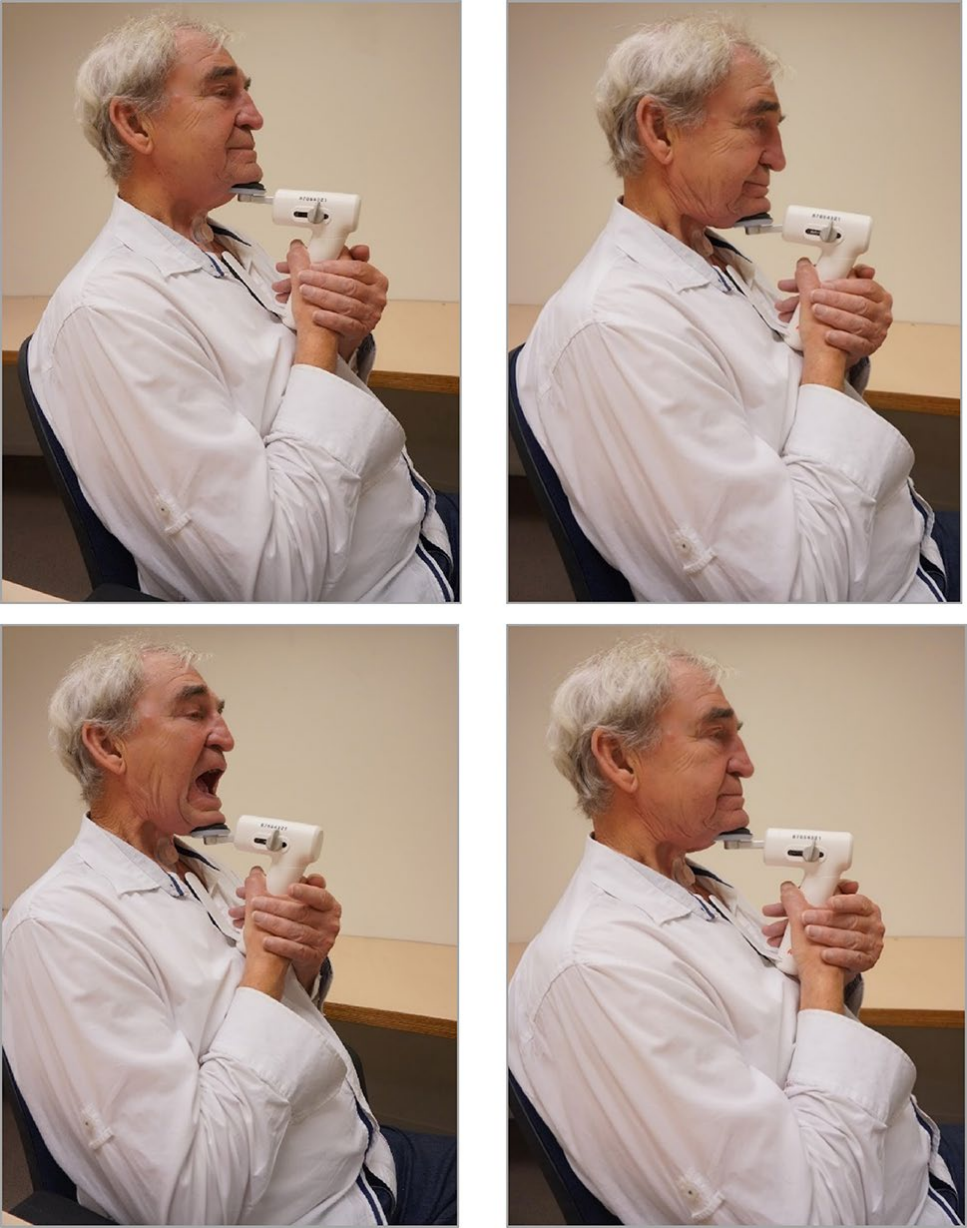
The Swallow Exercise Aid (SEA 2.0) exercises (printed with permission of the participant). Top left: start position, Top right: Chin Tuck Against Resistance (CTAR), Bottom left: Jaw Opening Against Resistance (JOAR), Bottom right: Effortful Swallow Against Resistance (ESAR) with 50% of maximum range of motion

The six-week exercise protocol (identical to the one published by Kraaijenga et al., 2015) of the CTAR and JOAR exercises consisted of an isokinetic and isometric part. During the isokinetic part, the participant was asked to perform the exercise 30 times. During the isometric part, the participant performed the exercise three times for 60 s, with at least 60 s of rest between the sets. The ESAR was performed ten times consecutively after another 60 s of rest. The total duration of the exercises was estimated to be 15–20 min per session. The exercises are displayed in Fig. 4.

All participants were instructed to perform the SEA 2.0 exercises three times a day, seven days a week, for six weeks in total. They received a written instruction sheet with pictures before starting their ‘six-week training period.’ Moreover, they received daily WhatsApp messages as a reminder to perform the exercises and to ensure that they could quickly get in touch if necessary. Once every one or two weeks, participants came to the hospital for a check-up. During this check-up, the clinician checked if they were doing well, whether he or she was performing the exercises correctly, whether they could increase the training resistance of the SEA 2.0 with one step, and to evaluate the Chin Tuck and Jaw Opening strength with the MicroFET. Participants were instructed to decrease the resistance or stop the exercises if they felt discomfort or pain in the chest, chin, neck, or in/around their temporomandibular joint during or after the exercises.

### Feasibility and Adherence

The feasibility of training with the SEA 2.0 and the content of the multidimensional assessment program was assessed using a study-specific questionnaire at the T1 measurements (Online Appendix: Questionnaire 1). To assess the adherence to the SEA 2.0 exercises, participants received a paper exercise logbook before their six-week training period started. All participants were asked to fill in the logbook after every training session and to keep the logbook up to date (Online Appendix: Fig. 14). Exercise adherence was considered good if participants completed for at least 70% of all training sessions.

Patients were evaluated again eight weeks later (T2). For the T1-T2 period, participants received a study-specific questionnaire (Online Appendix: Questionnaire 2) in which they were asked if they had trained with the tool during their eight-week resting period. Other questions were if they experienced differences in swallowing and strength and whether they planned to continue training with the SEA 2.0.

### Statistical Analysis

All statistical analyses were performed in R (version 4.2.1). [53] Descriptive statistics were used to characterize the sample. Continuous variables were summarized using median and range. Inter- and intra-observer reliability were assessed using the Two-way random effects model Intraclass Correlation Coefficient (ICC) and interpreted as follows: < 0.30 (negligible), 0.30 to < 0.50 (low), 0.50 to < 0.70 (moderate), 0.70 to < 0.90 (high), and ≥ 0.90 (very high, positive, or negative) correlation.

Linear mixed-effects models (LME) were used to summarize the changes over time of continuous outcomes. Time was used as a categorical variable. We used a random intercept per patient to account for the repeated observations and added a random slope if this improved model fit according to the AIC. This model takes missing outcome data (NA) into account. The estimated marginal means from the model, with a corresponding 95% confidence interval, were plotted along with the individual data. For discrete ordinal outcomes, we present the number and percentages patients improving, worsening, or staying the same.

## Results

All participants completed the baseline (T0) measurements, the six-week training protocol, and the subsequent measurements (T1). Nineteen participants completed the measurements eight weeks later (T2), while one participant had to decline due to medical conditions (not related to this study).

### Participant-Reported Outcomes

#### Laryngectomy Dysphagia Complaints Inventory

Table 2 displays the baseline LDCI score at T0, where a total of 285 complaints were noted as ‘yes’ or ‘present.’ After six weeks of training, these complaints reduced to 215 at T1 (see Table 3) and remained more or less stable at T2, except for liquid wash and chin edema. With regard to the need for using liquids to wash the food down (question 12), of the ten patients that did not need it anymore at T1, five relapsed at T2. With regard to the chin edema (question 26), this returned in all ten patients were it had improved.

**Table 3:**
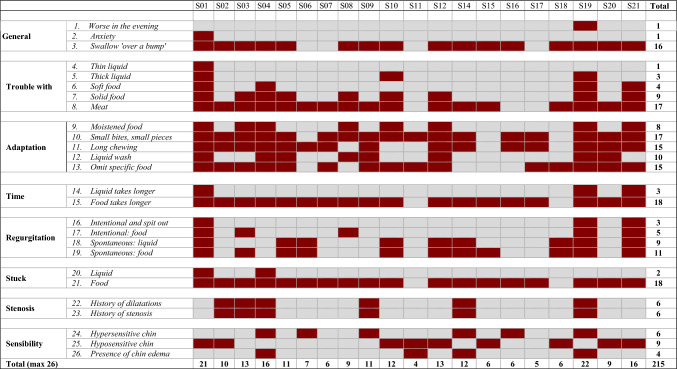
The Laryngectomy Dysphagia complaints inventory—T1 assessment after six weeks of training

*Dark red* Dark red means that the complaint is present, *gray* means that the complaint is absent

#### Subjective Perspectives on General Health

The participant-reported data on general health are shown in the Online Appendix Figs. 15 and 16. No changes were observed on the EQ-5D5-L Index and VAS scores from T0 to T1 and T2.

#### Subjective Perspectives on Swallowing Function and Oral Intake

**Fig. 5:**
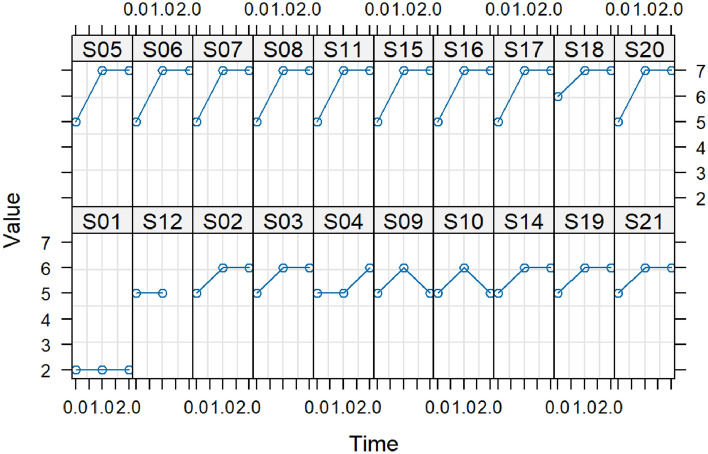
The Functional Oral Intake Scale (FOIS) per participant assessed oral intake status and type of diet. The FOIS is a reliable and validated tool that ranges from 1 to 7, with Nothing by mouth (1) and No oral restriction (7)

At baseline, one participant (5%) used a feeding tube (FOIS 2), while 19 (95%) had an oral diet with varying consistencies but needed special preparation (FOIS 5 and 6). Fifteen participants (75%) maintained higher FOIS scores at T1 and T2. Two (10%) improved temporarily at T1 but returned to baseline at T2, and three (15%) stayed constant, with one improving at T2 (see Fig. 5).

**Fig. 6:**
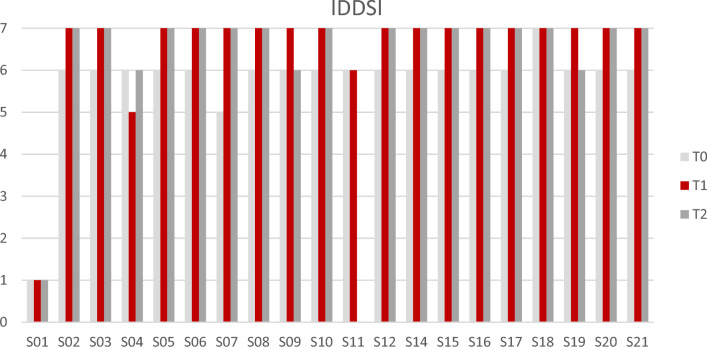
The International Dysphagia Diet Standardization Initiative (IDDSI) gives an overview of the type of diet per participant. The levels range from 0 to 7, with Thin liquid (0) and Normal solid or easy to chew consistencies (7)

Fourteen participants maintained their IDDSI levels consistently. Three kept a constant level, two briefly increased at T1 but returned to baseline at T2, and one briefly decreased at T1 but returned to baseline at T2 (see Fig. 6).

**Fig. 7:**
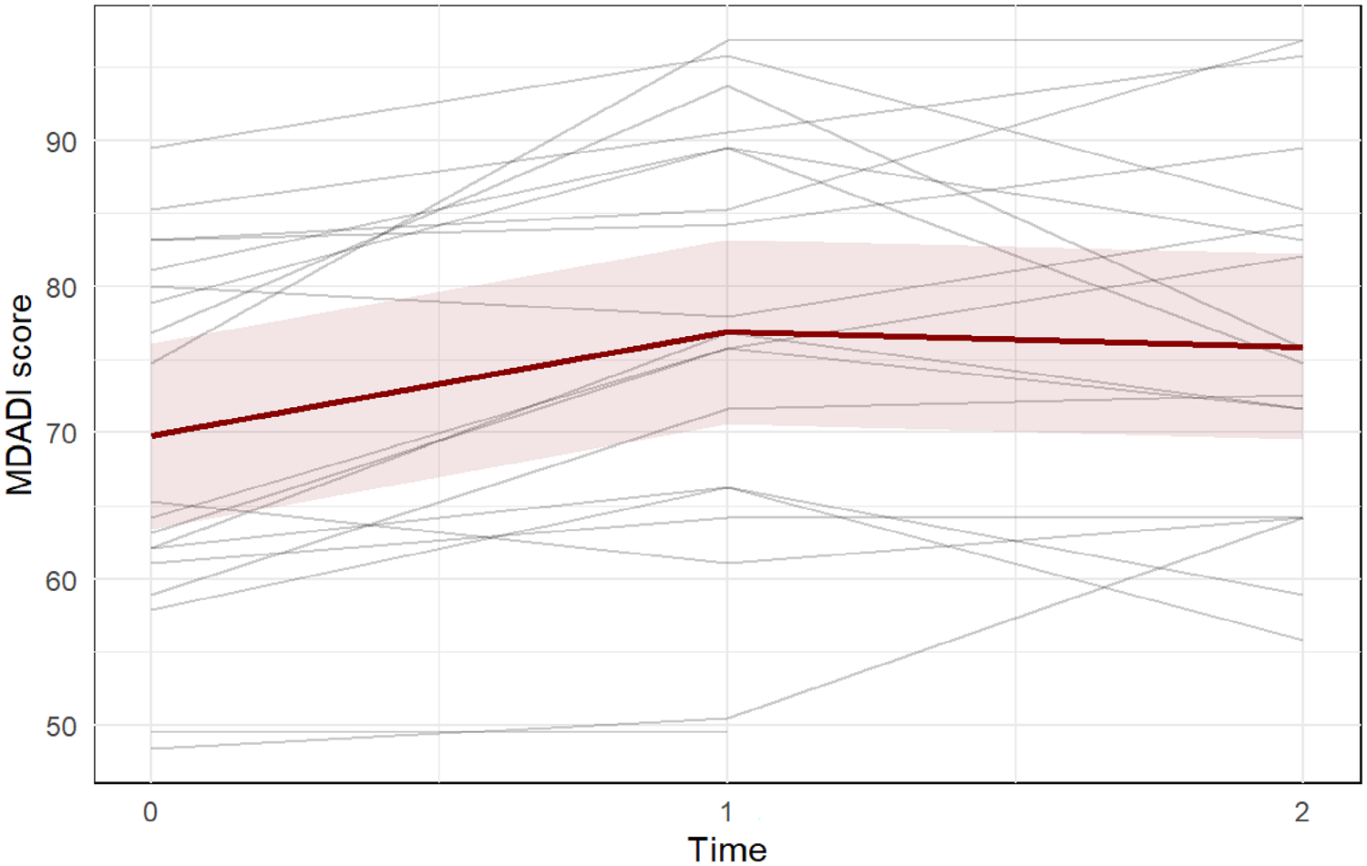
The MD Anderson Dysphagia Inventory (MDADI) questionnaire evaluates the impact of dysphagia on the quality of life. Total scores range from 20 (extremely low functioning) to 100 (high functioning). Each gray line represents one participant, while the red line represents the predicted marginal mean from the LME model, with the pink shading indicating the 95% confidence interval

**Fig. 8:**
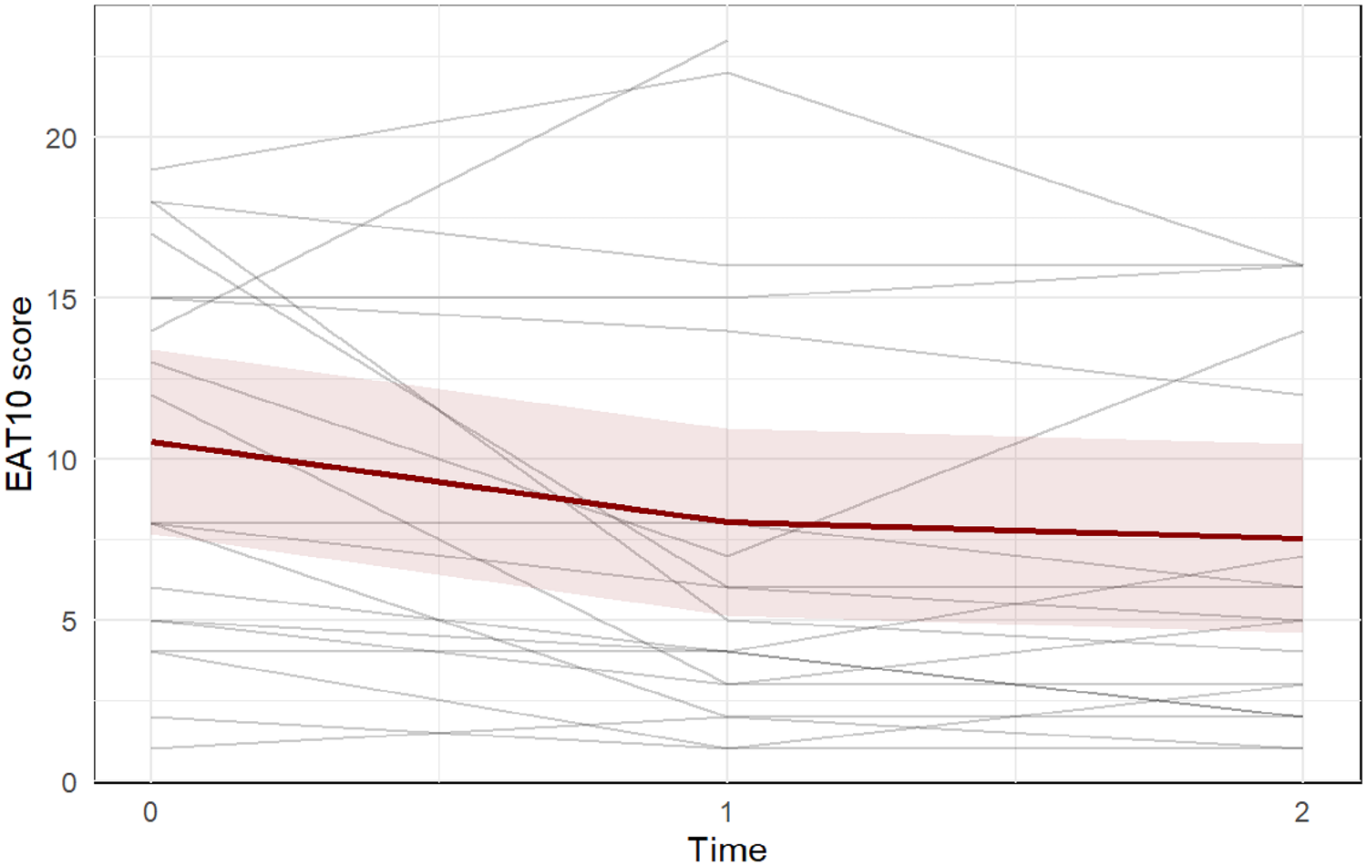
The Eating Assessment Tool (EAT-10) questionnaire includes ten questions that assess the initial dysphagia severity. A score of three or more is considered abnormal. Each gray line represents one participant, while the red line represents the predicted marginal mean from the LME model, with the pink shading indicating the 95% confidence interval

**Fig. 9:**
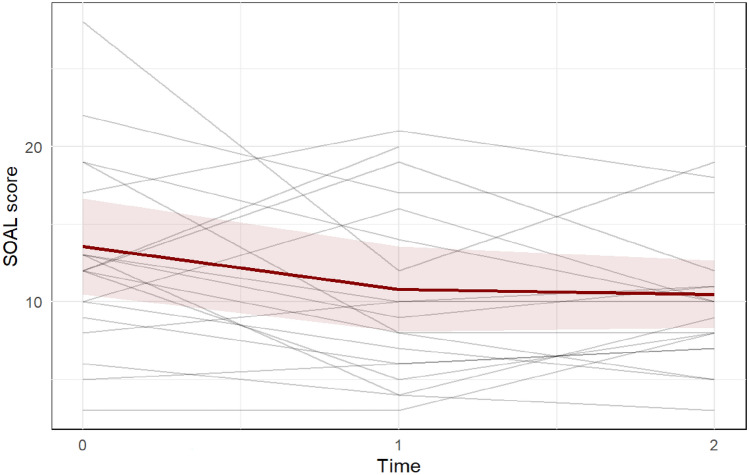
Swallowing Outcomes after Laryngectomy (SOAL) questionnaire focuses on swallowing problems, specifically for laryngectomized patients. Lower scores indicate fewer problems and better self-reported overall swallowing function. [41] Each gray line represents one participant, while the red line represents the predicted marginal mean from the LME model, with the pink shading indicating the 95% confidence interval

The MDADI improved with 7.1 points from 69.8 (95%CI 63.4–76.1) at T0 to 76.9 (95%CI 70.6–83.2; Cohen’s D 0.58) at T1 and was largely maintained at T2 with 75.9 (95%CI 69.5–82.2) see Fig. 7. The EAT-10 decreased/improved from 10.6 (95%CI 7.7–13.4) at T0 to 8.0 (95%CI 5.1–10.9) at T1 and was largely maintained at T2 with 7.5 (95%CI 4.6–10.5), see Fig. 8. The SOAL slightly decreased/improved from 13.6 (95%CI 10.8–16.3) at T0 to 11.0 (95%CI8.2–13.8) at T1 and maintained at T2 with 10.6 (95%CI 7.8–13.4), see Fig. 9.

### Objective Measurements

#### Body Length, Body Weight, and BMI

No changes were observed on body weight and BMI between from T0 to T1 and T2, see Online Appendix Figs. 17 and 18. At T0, body weight was 89.7 kg (95%CI 82.6–96.7) and BMI scores were 27.9 (95%CI 26.1–29.8).

#### Swallowing Assessment

**Fig. 10:**
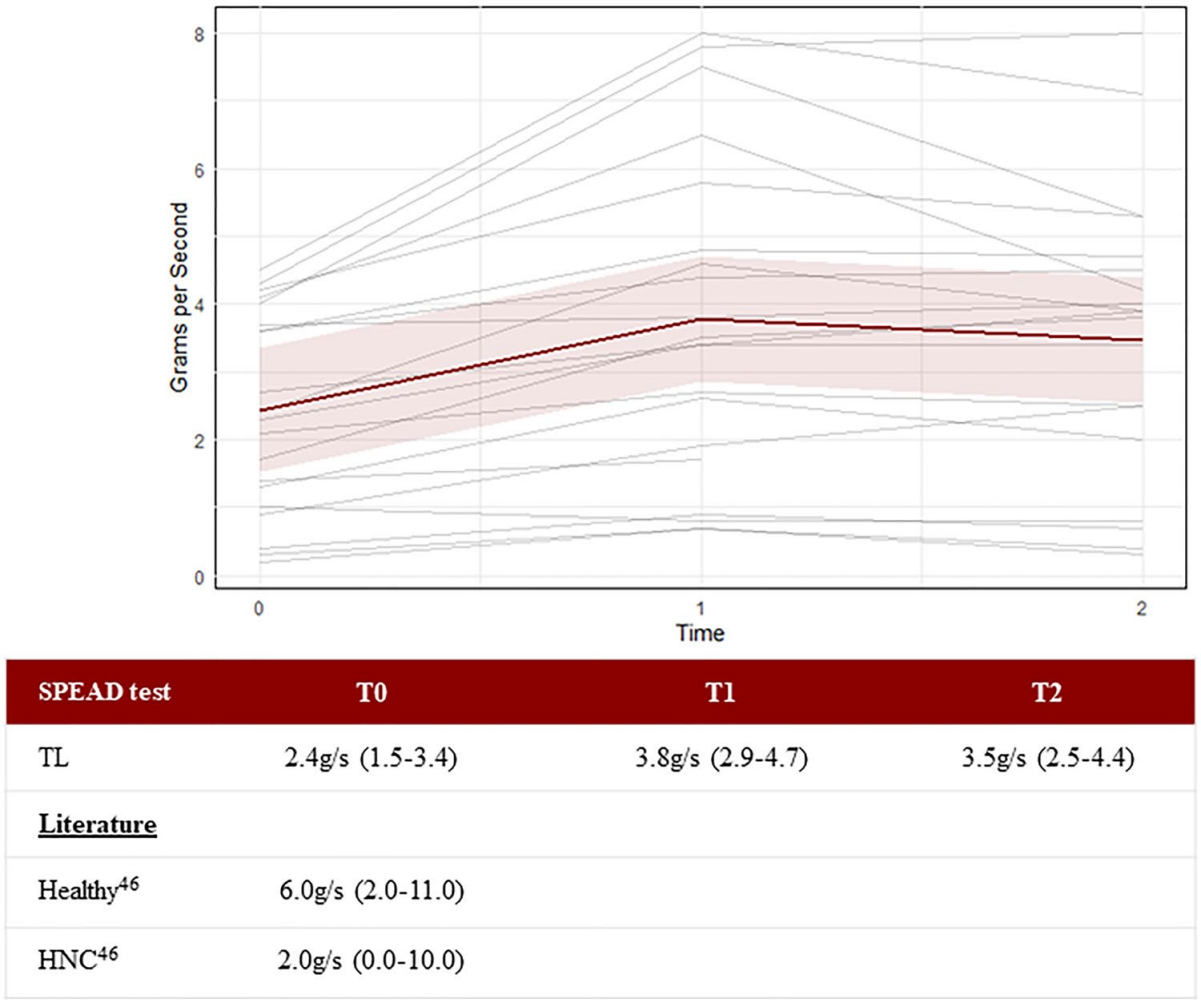
The Swallowing Proficiency for Eating and Drinking (SPEAD) test, which measures the swallowing capacity in grams per second. Each gray line represents one participant, while the red line represents the predicted marginal mean from the LME model, with the pink shading indicating the 95% confidence interval

The intra-rater reliability for the SPEAD test was 0.946; and the interrater reliability was 0.961. The SPEAD test increased from 2.44 g/s (95%CI 1.51–3.36) at T0 to 3.78 g/s (95%CI 2.85–4.70) at T1 and this improvement was largely maintained at T2 with 3.47 g/s (95%CI 2.54–4.39), see Fig. 10.

**Fig. 11:**
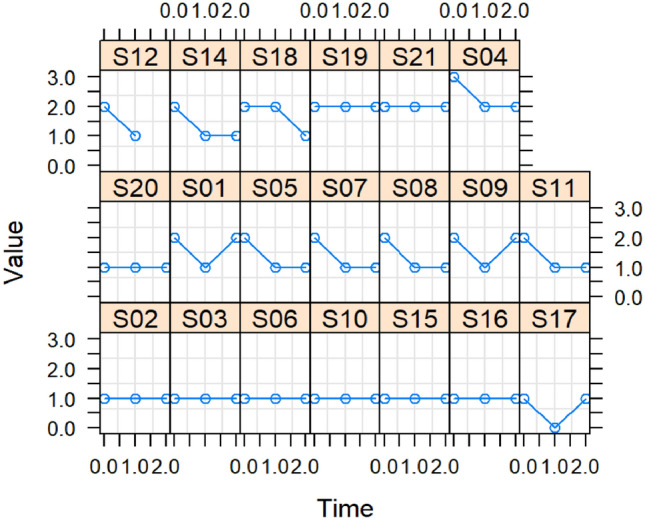
The Dynamic Imaging Grade of Swallowing Toxicity (DIGEST) scores per participant based on the percentage of residue in the neopharynx after the initial swallow. The overall DIGEST Grade is based on the interaction between the Safety Grade (scored as 0 in our participants) and the Efficiency Grade (scored from 0 to 4) and can be interpreted as the severity of dysphagia (0 = Safe & Efficient, 1 = Safe and Mildly Inefficient, 2 = Safe and Moderately Inefficient, 3 = Safe and Severely Inefficient, 4 = Safe and Profoundly Inefficient). A lower score can be interpreted as better swallowing efficiency and reduced residue in the neopharynx

For the DIGEST test, the intrarater reliability was 0.789, and the interrater reliability was 0.865. The percentage residue on the VFSS decreased in 10 participants between T0 and T1. In six participants, this effect was maintained at T2, while two participants had a relapse. Nine participants remained stable over time, see Fig. 11.

During VFSS at baseline, no leakage through or around the voice prosthesis was observed. In nine participants (45%), a pseudovallecula or pseudoepiglottis was observed. Stenosis was seen in six participants (30%). Two participants (10%) showed nasal regurgitation, six (30%) oral regurgitation, and 19 (95%) pharyngeal regurgitation. In 15 participants (75%), pre-swallow posterior spilling was observed, especially in the 40 cc thin liquid (IDDSI 0) and solid bolus (IDDSI 7). Oral residue was seen in five participants (25%), and pharyngeal residue in all participants (100%) in all consistencies. Fourteen participants (70%) needed a liquid wash after extremely thick liquid (IDDSI 4), and 15 participants (75%) after solid intake (IDDSI 7).

#### Mouth Opening Assessment

None of the participants showed trismus and no effects were observed on mouth opening, see Online Appendix Fig. 19.

#### Tongue Strength and Swallowing Muscle Assessment

The baseline anterior tongue strength, measured with IOPI, was 50.6 kPa (95%CI 45.1–56.1) and no changes were observed over time, see Online Appendix Fig. 20.

**Fig. 12:**
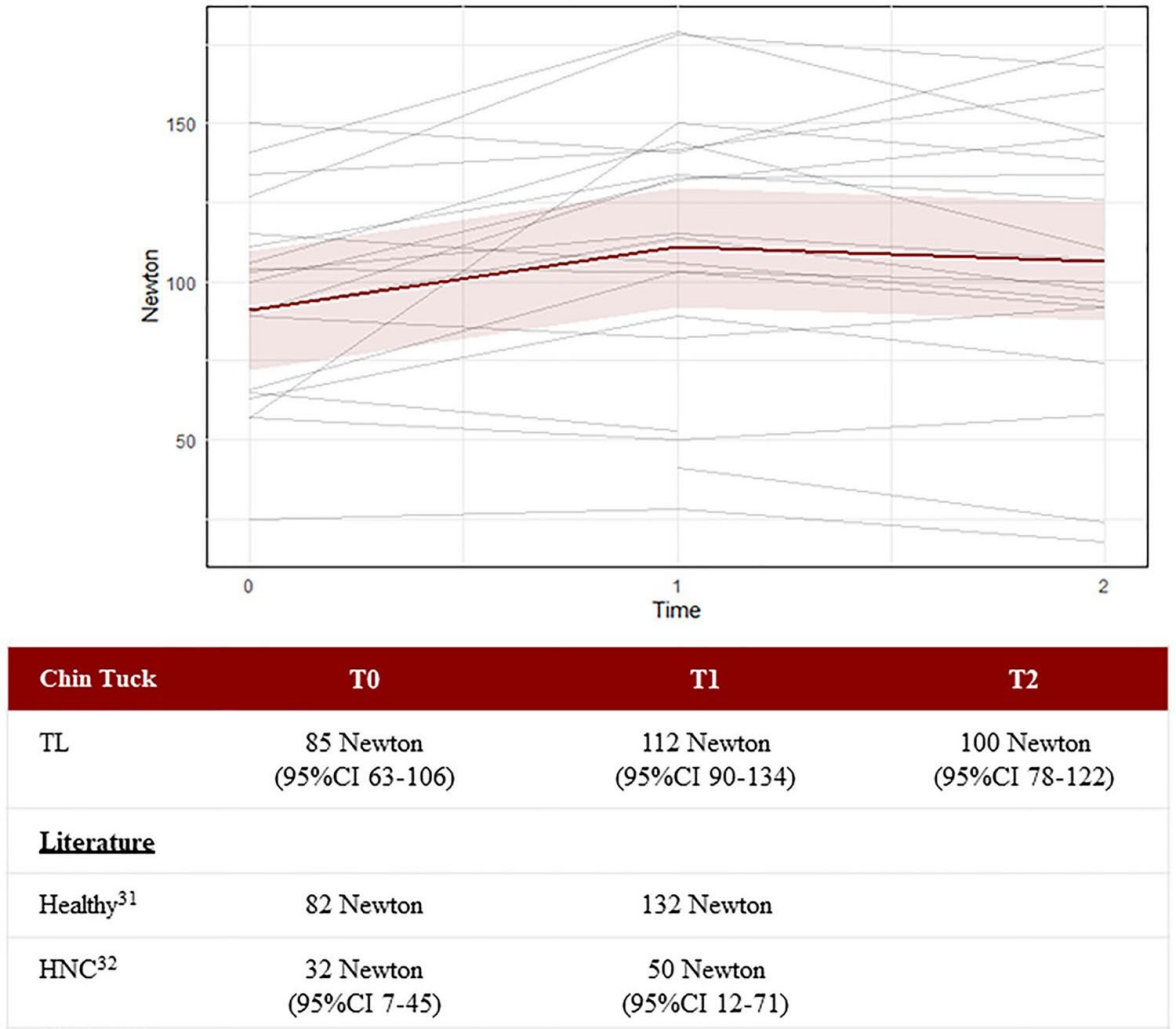
The Chin Tuck strength assessment measured in Newton and displayed per person. Each gray line represents one participant, while the red line represents the predicted marginal mean from the LME model, with the pink shading indicating the 95% confidence interval

The Chin Tuck strength increased from 84.5 Newton (95%CI 62.8–106.2) at T0 to 111.9 Newton (95%CI 90.2–133.6) at T1 and decreased at T2 to 99.7 Newton (95%CI 77.9–121.6), see Fig. 12.

**Fig. 13:**
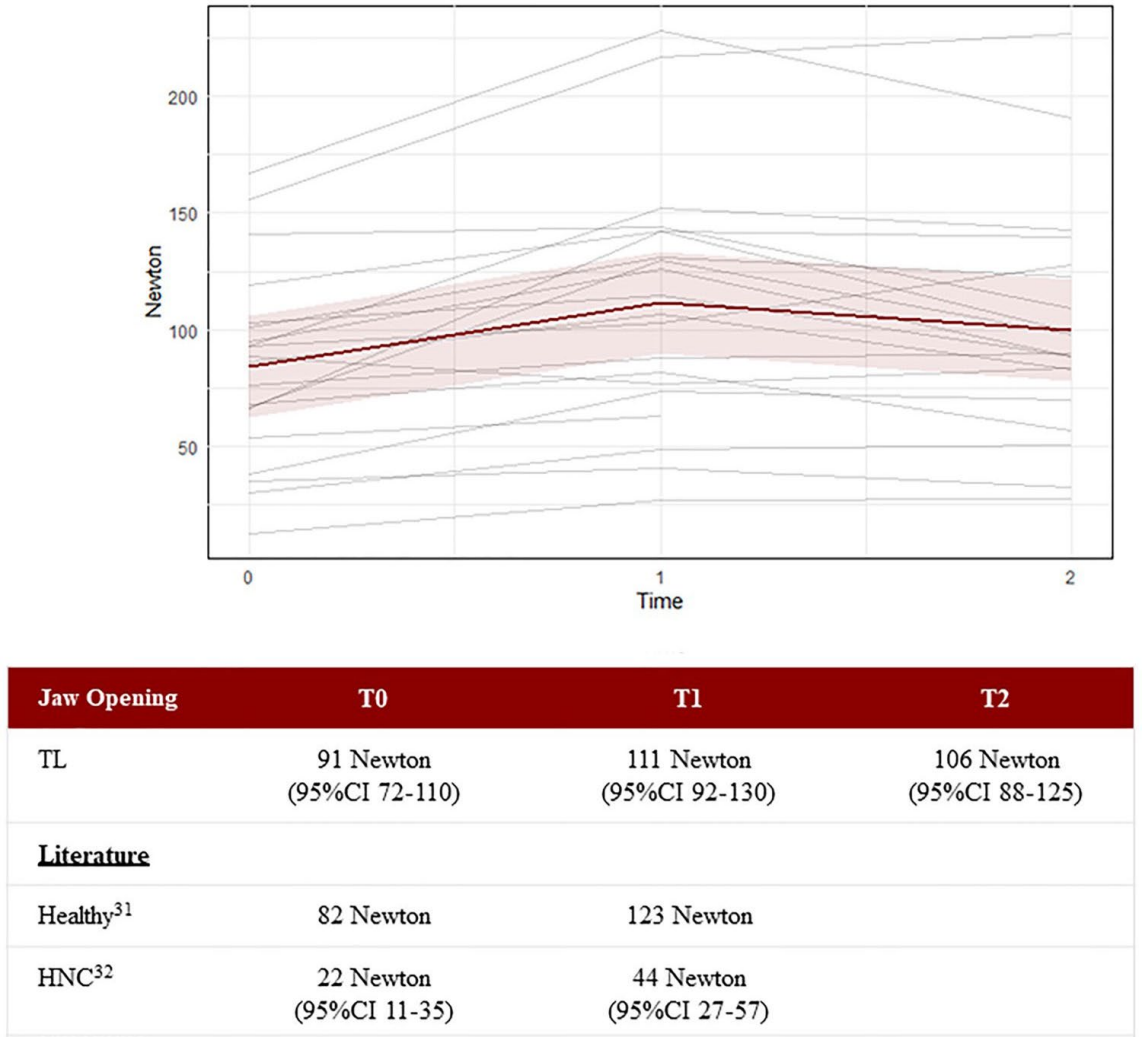
The Jaw Opening strength assessment measured in Newton and displayed per person. Each gray line represents one participant, while the red line represents the predicted marginal mean from the LME model, with the pink shading indicating the 95% confidence interval

The Jaw Opening strength increased from 90.8 Newton (95%CI 71.9–109.6) at T0 to 110.9 Newton (95%CI 92.1–129.6) at T1 and decreased at T2 to 106.4 Newton (95%CI 87.5–125.2), see Fig. 13.

### Swallow Exercise Aid (SEA) 2.0

All participants were able and willing to use the SEA 2.0 device. The SEA 2.0 device did not impact the tracheostoma. Respiration was stable during execution of the CTAR, JOAR, and ESAR exercises (see Fig. 4).

Table 4 shows the 1RM calculated for the Chin Tuck and Jaw Opening strength in Newton. During the try-out session, the participants found the isometric part of the exercise more strenuous than the isokinetic part. Therefore, the CTAR and JOAR exercises were divided into two sub-exercises: ‘isokinetic’ and ‘isometric.’

**Table 4:** Individualization of the start level of the SEA 2.0 for CTAR and JOAR exercise

	CTAR						JOAR					
	Chin Tuck Muscle Strength			Load Graph	Patient adjusted start level		Jaw Opening Muscle Strength			Load Graph	Patient adjusted start level	
	1RM (Newton)	60–75%	40–60%	Start level (%1RM)	Isokinetic (%1RM)	Isometric (%1RM)	1RM (Newton)	60–75%	40–60%	Start level (% 1RM)	Isokinetic (%1RM)	Isometric (%1RM)
S01	112	67–84	45–67	6 (80)	4 (45)	3 (36)	119	71–89	47–71	6 (76)	4 (42)	3 (34)
S02	97	58–73	39–58	5 (62)	4 (52)	3 (41)	109	65–82	44–65	5 (55)	4 (46)	3 (37)
S03	107	64–80	43–64	5 (56)	4 (47)	3 (37)	116	69–87	46–69	6 (78)	3 (35)	2 (26)
S04	77	46–58	31–46	4 (65)	4 (65)	4 (65)	93	56–69	37–56	5 (65)	3 (43)	3 (43)
S05	41	25–35	16–25	2 (73)	3 (98)	2 (73)	64	38–48	26–38	3 (63)	3 (63)	2 (47)
S06	179	108–134	72–107	7 (67)	5 (33)	5 (33)	153	92–114	61–92	7 (79)	5 (39)	4 (33)
S07	145	87–109	58–87	6 (62)	4 (34)	4 (34)	166	100–125	66–100	7 (72)	5 (36)	4 (30)
S08	78	47–59	31–47	4 (64)	2 (38)	2 (38)	69	41–52	28–41	4 (73)	2 (44)	2 (44)
S09	124	74–93	49–74	6 (73)	5 (49)	5 (49)	105	63–78	42–63	5 (57)	4 (48)	4 (48)
S10	192	115–144	77–115	7 (63)	5 (31)	5 (31)	137	82–103	55–82	6 (66)	4 (36)	3 (29)
S11	111	66–83	44–66	5 (54)	4 (45)	4 (45)	105	63–79	42–63	4 (57)	3 (38)	3 (38)
S12	57	34–43	23–34	3 (70)	2 (52)	2 (52)	67	40–50	27–40	4 (75)	3 (60)	3 (60)
S14	29	17–22	12–17	1 (69)	2 (104)	1 (69)	58	35–43	23–35	3 (69)	2 (52)	1 (35)
S15	90	53–67	36–54	5 (67)	4 (56)	3 (45)	93	56–70	37–56	5 (65)	3 (43)	3 (43)
S16	76	45–57	30–45	4 (66)	3 (53)	2 (40)	98	59–73	39–59	5 (61)	2 (31)	2 (31)
S17	68	41–51	27–41	4 (74)	3 (59)	2 (44)	61	36–45	24–36	3 (66)	3 (66)	2 (50)
S18	86	52–65	35–52	5 (70)	3 (46)	2 (35)	111	66–83	44–66	5 (54)	3 (36)	2 (27)
S19	35	21–26	14–21	1 (57)	1 (57)	1 (57)	x	x	x	1 (x)	1 (x)	1 (x)
S20	115	69–86	46–69	5 (52)	3 (35)	2 (26)	146	88–110	58–88	6 (74)	2 (33)	1 (25)
S21	25	15–19	10–15	1 (80)	1 (80)	1 (80)	28	17–21	11–17	1 (73)	1 (73)	1 (73)

*1RM* 1 Repetition Maximum, *CTAR* Chin Tuck Against Resistance, *JOAR* Jaw Opening Against Resistance, *x* missing

During the try-out session, one participant could start at the 60–75% level of the isokinetic CTAR 1RM, sixteen participants started below 60%, and three at a level above 75% of the 1RM measured on the MicroFET. For the isometric CTAR exercise, three participants could start at approximately 60–75%, sixteen participants below 60%, and one at a level above 75%. For the isokinetic JOAR, four participants could start at levels between 60 and 75%, and the other 15 thought the resistance was too strenuous and started below 60%. One participant could not complete the measurement due to a hypersensitive chin, because of an extreme cough reflex and she started at level one. For the isometric JOAR, two participants could start at 60–75% and 17 had to start below 60%. The same patient with the cough reflex also started the isometric JOAR at level one.

### Feasibility and Adherence

All participants completed the six-week training protocol and kept their logbook up to date. Nineteen participants completed 70% or more of the training program, resulting in an adherence of 95%. One participant (S12) felt that the training was too intense and decided to train less. The median number of training days was 42 (range 22–42). The self-reported total duration of each session was between the 20 and 35 min. The training resistance per participant per week per exercise can be found in the Online Appendix Figs. 21–25.

At T1, eight participants reported the exercises with the SEA 2.0 as “bit unpleasant”, eight participants as “neither pleasant nor unpleasant”, two participants as “a bit pleasant”, and two participants as “very pleasant”. After a median of 6 days (range 1–14), participants were used to all exercises. Reported problems were temporary muscle spasm in the jaw and cheek muscles (*N* = 3), accidental tooth injury of the lip (*N* = 1), and headache after training (*N* = 2). Participants felt improvements in strength or swallowing after a median of 14 days (range 5–30). Eleven participants reported being willing to continue the exercise training if prescribed. Seven participants were probably willing to continue the exercises, and two participants were not willing to continue the exercises. The intensive measurement was accepted very well. Only the minority of the participants scored the assessment protocol as bit unpleasant (*N* = 3). The majority found the protocol neither pleasant nor unpleasant (*N* = 17).

At T2, two participants reported that they had continued the training despite the advice to have an eight-week rest period. One participant (S1) did not want to lose his gained strength, and another (S5) felt that her swallowing function and strength were decreasing. At T2, nine participants (45%) reported that their swallowing function decreased over the past eight weeks, and five participants (25%) felt that their edema increased again during their rest period. Thirteen participants (65%) reported that they would start again with training with the SEA 2.0 after the obligatory rest period of the study protocol.

## Discussion

The aims of this study were to assess the specific nature and extent of dysphagia in a group of laryngectomized patients with self-reported swallowing problems, and the usability of the novel SEA 2.0 device as a rehabilitation tool, and, in a 6-week clinical trial, evaluate the feasibility and adherence of the training program, and explore the early and longer-term (8 weeks later) effects on swallowing function.

The outcomes of this study suggest that the training program is feasible and results in improvement in several relevant outcomes. All twenty participants were able to complete the full training program with the SEA 2.0. The adherence with 95% of the participants adhering to the protocol was excellent, and the multidimensional assessment showed improvements in the swallowing-focused PROMs (MDADI and EAT-10), in CTAR, JOAR, and ESAR strengths, and in the objective parameters, swallowing capacity (SPEAD test) and pharyngeal residue clearance (VFSS analysis with the DIGEST). After eight weeks of rest, the outcomes of the PROMs and objective swallowing assessments were slightly reduced, but still significantly and relevantly better than at baseline.

To our knowledge, this is the first study that investigated the possibility to train head and neck muscles involved in swallowing after a laryngectomy. Before the start of the six-week exercise program, the multidimensional assessment showed that dysphagia in this selected group of laryngectomized patients has a serious impact on their quality of life. Interestingly, however, despite problems with bolus propulsion, prolonged mealtimes and limited or adjusted diet tolerance, the BMI in this patient cohort appeared to be more or less normal. The normal BMI values might be an explanation why, unlike the more obvious voice and airway issues after TL, dysphagia has not received similar rehabilitation attention after this surgery. General health status in this selected group of dysphagia patients was also relatively good. Most participants scored “normal health” values (according to the reference score of 0.839) with an index of 0.850 (%CI 0.760–0.930) and a median VAS score of 78 (95%CI 73–84) at the EQ-5D-5L [33]. This relatively high level of QOL is in accordance with the earlier reports [54].

Possible effects of strength on dysphagia were explored. The tongue strength (IOPI) was only performed anteriorly because most participants had a gag reflex by trying the posterior position. The tongue strength of 50.6 kPa (95%CI 45.1–56.1) was comparable with the tongue strength found by Clark et al. (2012) in healthy older subjects (≥ 60 years) 51 kPa (SD 12.97), and with the median tongue strength (57.4 kPa) of the healthy individuals of the study of Kraaijenga et al. (2015) [31, 51]. The Chin Tuck strength of our participants (84.5 Newton) was comparable with the median strength of healthy seniors (82.0 Newton) and quite a lot higher than the median strength of post-(C) RT Head and Neck cancer patients (31.5 Newton) [31, 32]. In terms of the Jaw Opening strength assessment, the median strength of the laryngectomized participants (90.8 Newton) was somewhat higher than the median strength of healthy seniors (82.3 Newton), but substantially higher than that of Head and Neck cancer patients after (C) RT (21.5 Newton) [31, 32]. A possible explanation could be that laryngectomized participants train their muscles every time they swallow, when trying to overcome their increased pharyngeal resistance due to the altered anatomy and physiology, without having to be afraid of aspiration [15].

During the try-out session with the SEA 2.0, all participants were able to handle the device and in none of them, the stoma was blocked. Lymphedema of the chin and the mentioned hypo- and hypersensitivity of the chin did not seem to be a problem in performing exercises with the SEA 2.0. As it was unclear how 1RM measurements with the MicroFET would translate to training load with the novel SEA2.0, we adopted a pragmatic approach to determining training load. Starting at a high load, and then gradually reducing the load to self-reported acceptable resistance at 30 repetitions, resulted in an exercise prescription reflecting 40–60% of the 1RM for the majority of patients. Such a training load has been recommended before to improve the muscle function in senior adults [55]. This correlates very well the patient’s own perception as can be seen in Table 4. For clinical application, a simpler approach could be used in which the 1RM is determined directly with the SEA 2.0 device, and the training load set to fall between 40 and 60% of this load. Progression could then be based on the concept of “repetitions in reserve”, where the load is progressed if the there is no local exhaustion after the 30 repetitions (i.e., the patient would be able to do more repetitions) [56].

After the six-week training period, the Chin Tuck strength assessment showed an increase of 27.4 Newton (T0 to T1), and the Jaw Opening strength assessment showed an increase of 20.1 Newton (T0 to T1). These gains were lower than those in healthy individuals (38.5 Newton for Chin Tuck and 52.1 Newton for Jaw Opening) but higher than in head and neck cancer survivors (18.0 Newton for Chin Tuck and 22.0 Newton for Jaw Opening) [31, 32]. This clearly shows that after total laryngectomy training with the SEA 2.0 can increase CTAR and JOAR strengths.

On the swallowing-focused PROMs, significant improvements were found at T1 on the MDADI, SOAL, and EAT-10. Although the score on the MDADI significantly increased with 7.1 points to 76.9 (95%CI 70.6–83.2; Cohen’s D 0.57), which according to a recent letter to the editor by McDowell et al. is clinically relevant [57, 58]. The EAT-10 and SOAL scores both decreased with 2 points. The SOAL questionnaire is currently under investigation for its validity in Dutch and might therefore not be reliable at this moment.

The shortage of validated swallowing assessment tools for this patient category was tried to overcome with the LDCI (Table 2 and 3) based on the structured interview taken with every patient. It showed that all participants had to adapt their food and eating habits. For instance, all participants omitted specific food or had to use a liquid wash. Notably, after six weeks of training, there were notable shifts in the adaptation strategies employed by the participants.

The FOIS and IDDSI scores support these findings. On the FOIS, level 5 (Total oral intake with multiple consistencies but requiring special preparation or compensation) and level 6 (total oral diet with multiple consistencies without special preparation but with specific food limitations) were the most common scores. The IDDSI scores showed that all participants were able to drink thin liquid (IDDSI 0) and that the majority of the participants adapted their food by making it Minced and Moist (IDDSI 5) or Soft and Bite-sized (IDDSI 6) at baseline. After the six-week training period, most FOIS scores increased and more participants were able to consume Soft and Bite-sized (IDDSI 6) and Normal or Easy to Chew solids (IDDSI 7). And after eight weeks of rest, these outcomes were comparable (see Figs. 5 and 6).

The analysis of the VFSS with the DIGEST showed that the percentage residue on the VFSS decreased in 10 participants between T0 and T1. In six participants, this decrease maintained at T2, and two participants had a relapse. Nine participants remained stable over time, see Fig. 11. With these results, it might be reasonable to assume that increasing the strength of the (remained or reattached) muscles might positively affect the clearance of the neopharynx. In view of swallowing time being a dominant factor, this coincides well with the improvements in swallowing capacity measured with the SPEAD test, from a median of 2.44 g/s (95%CI 1.51–3.36) at baseline to 3.78 g/s (95%CI 2.85–4.70) at T1. From the studies of Karsten et al., we have some reference values for the ‘normal’ SPEAD rate values for healthy participants (median 6, range 2–11) and for Head and Neck cancer patients with dysphagia (median 2, range 0–10) [46]. The median SPEAD rate in our cohort of 2.44 g/s at baseline is comparable to the head and neck dysphagia reference group. In other words, the laryngectomized participants need as much time as the Head and Neck cancer patients treated with (C) RT to swallow, eat, and drink [46]. Although the improvement to 3.78 g/s at T1 is still not comparable to the values observed in healthy individuals by Karsten et al., most participants noticed improvements.

The reassessment after eight weeks of rest (T2) showed interestingly only slight decreases of the gains, although one has to keep in mind that two participants continued exercising, because they did not want to lose the functional improvements. The outcomes of the swallowing-focused PROMs (EAT-10 and MDADI) were comparable to those at T1. The SPEAD rate decreased only slightly, and the outcomes on the DIGEST stayed comparable in most participants. Strength on the Chin Tuck and Jaw Opening decreased, but was still higher than the baseline. Thus, although all improved measurements stayed higher than those at baseline, this suggests that maintenance therapy should be considered to stabilize or even further improve swallowing function and strength.

The current sample size does not allow for meaningful subgroup analysis, since the resulting small subgroups would likely be unbalanced on several other factors influencing the outcome.

Not every participant showed improvements on the PROMs and objective assessments, though. For instance, participant S04 suffered from a persistent stenosis and practicing with the SEA 2.0 did not prevent the need for another dilatation. At T1, his stenosis was so severe that he had to adjust his oral intake (FOIS and IDDS scores) to prevent esophageal blockage. After receiving dilatation between T1 and T2, his outcomes improved at T2. It remains uncertain whether his participation in the study and strength exercises also played a role in these improvements. Another participant, S12, found the training protocol too intensive and decided to train less, resulting in less improvements in any of the measurements. At the time of T1, S15 was recovering from a flu and therefore did not improve in all measurements. Later, he was diagnosed with metastasis, a condition unknown during the study but possibly relevant at that time. Participant S14 reported severe headaches and muscle cramps after training. Despite this, she insisted to complete the full training protocol. To the training load, therefore, it was decreased which resolved the complaints (see Online Appendix Figs. 21–25). Despite her decreased training levels, she did show progress during the assessments.

### Limitations and Strengths

The current study focused on examining the swallowing function in a specific group of laryngectomized individuals, all of whom experienced dysphagia, and almost one in three also had experienced stenosis with dilatation. Consequently, it is important to note that the findings of this study may not be directly applicable to the entire laryngectomized population.

The study protocol achieved an excellent 95% adherence rate. This percentage may be attributed to several factors, including participants being instructed to maintain a logbook, maintaining daily communication with their clinician via WhatsApp, and attending regular check-ups every 1–2 weeks for assessments of Chin Tuck and Jaw Opening strength. This also gave participants valuable insights into their (two)-weekly progress.

Considering our adherence rate, we employed three methods to motivate participants: exercise instructions on paper, maintaining a logbook, daily WhatsApp contact, and bi-weekly hospital check-ups. However, this approach may not be practical for everyday situations. It’s essential to determine which method is most effective for long-term guidance. Additionally, insights into this matter may emerge from the outcomes of the ongoing PRESTO trial, currently being investigated by van Baudelet et al. [59]

Regarding the assessment of dysphagia, there is a limited availability of validated tools designed specifically for this patient population. Consequently, we utilized a study-specific structured interview (LDCI) and relied on dysphagia patient-reported outcome measures (PROMs) that had been validated for non-laryngectomized individuals. As a result, it is important to approach the interpretation of subjective assessments with some caution.

However, it’s worth noting that the objective measurements, such as the SPEAD test and DIGEST, demonstrated excellent interrater and intra-rater reliability. This suggests that the interpretation of these objective data is more robust.

### Future Research

The ability to apply progressive load in a systematic way using conventional swallow exercises is limited, contrary to the SEA 2.0, which offers this function, crucial for muscle strength improvement in any exercise regimen. Still, a comparative study with a control group without a device certainly is warranted.

To understand how strength exercises exactly target the remaining muscles after TL and such altered anatomy, more knowledge is needed about the physiology of swallowing after laryngectomy. Further imaging studies (e.g., MRI and/or High-Resolution (Impedance) Manometry) might be helpful in this respect. Furthermore, participants found the bootcamp-like training protocol of six weeks, seven days a week, and three times a day quite intensive. Despite the high adherence to the current program, dose-finding studies would be helpful to determine the minimal and optimal effective straining doses. Also, it would be useful to develop a minimal maintenance program. Another aspect worthwhile addressing is to objectify the suggestion of the patients that these exercises improve chin edema.

## Conclusion

This study explored the specific nature and extent of dysphagia in laryngectomized patients with self-reported dysphagia and showed the usability of the novel SEA 2.0 device as a rehabilitation tool for this patient population. All participants were able to complete the full 6-week training program with the SEA 2.0, and adherence was excellent. At the end of the training program, there were clinically relevant improvements in subjective swallowing outcomes, Chin Tuck and Jaw Opening strength, in the objective parameters swallowing capacity and pharyngeal clearance. After eight weeks of rest, all outcomes were slightly reduced, but still better than at baseline.

## Supplementary Information

Below is the link to the electronic supplementary material.

Supplementary file1 (DOCX 197 kb)

## Data Availability

The dataset generated and analyzed during the current study are available from the corresponding author upon reasonable request.
