# Correction to: Dysphagia After Total Laryngectomy: An Exploratory Study and Clinical Phase II Rehabilitation Trial with the Novel Swallowing Exercise Aid (SEA 2.0)

**DOI:** 10.1007/s00455-024-10711-4

**Published:** 2024-05-08

**Authors:** Marise Neijman, Frans Hilgers, Michiel van den Brekel, Rob van Son, Martijn Stuiver, Lisette van der Molen

**Affiliations:** 1 Department of Head and Neck Oncology and Surgery, The Netherlands Cancer Institute, Plesmanlaan 121, 1066 CX Amsterdam, The Netherlands; 2 Center for Quality of Life and Division of Psychosocial Research and Epidemiology, The Netherlands Cancer Institute, Plesmanlaan 121, 1066 CX Amsterdam, The Netherlands; 3 Amsterdam Center for Language and Communication (ACLC), University of Amsterdam, Binnengasthuisstraat 9, 1012 ZA Amsterdam, The Netherlands; 4 Department of Oral and Maxillofacial Surgery, Amsterdam University Medical Center, Meibergdreef 9, 1105 AZ Amsterdam, The Netherlands

**Correction to: Dysphagia** 10.1007/s00455-024-10673-7

In this article, the Figs. 12 and 13 had been inadvertently interchanged.

**Fig. 12:**
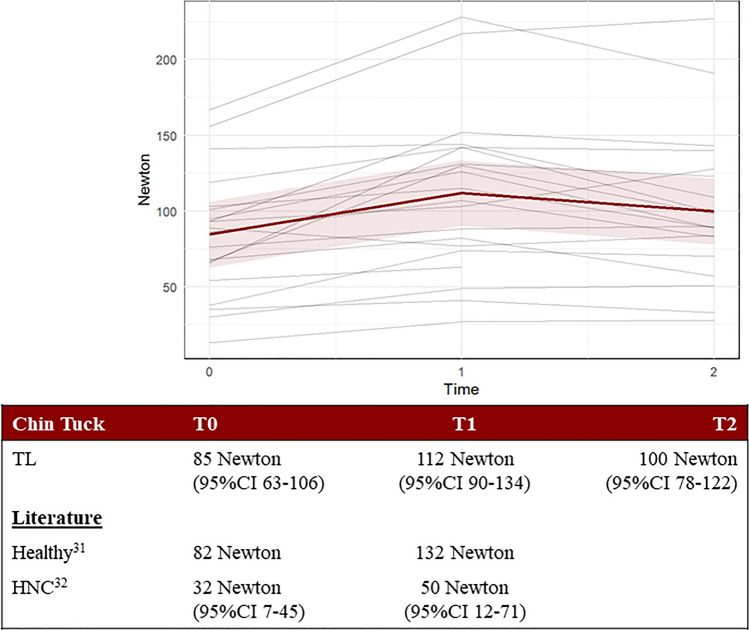
The Chin Tuck strength assessment measured in Newton and displayed per person. Each gray line represents one participant, while the red line represents the predicted marginal mean from the LME model, with the pink shading indicating the 95% confidence interval

**Fig. 13:**
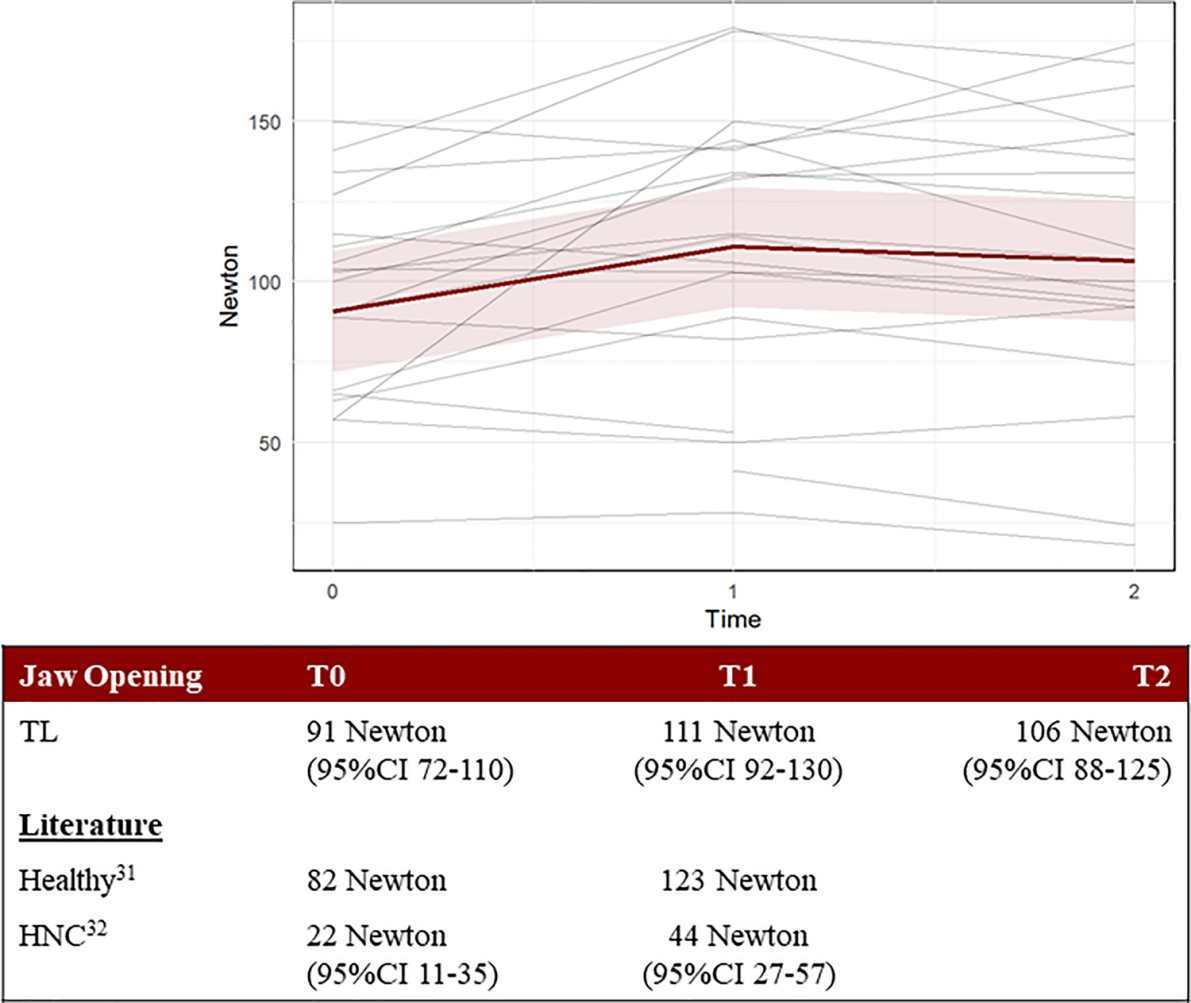
The Jaw Opening strength assessment measured in Newton and displayed per person. Each gray line represents one participant, while the red line represents the predicted marginal mean from the LME model, with the pink shading indicating the 95% confidence interval

The correct version of figures and captions are given below.

The original article has been corrected.

