# Supplementary material for: Dysphagia After Total Laryngectomy: An Exploratory Study and Clinical Phase II Rehabilitation Trial with the Novel Swallowing Exercise Aid (SEA 2.0)
Source: Dysphagia. 2024 Apr 1;39(5):916–36. doi: 10.1007/s00455-024-10673-7 (PMC11449961; doi:10.1007/s00455-024-10673-7)
Supplement: Supplementary file 1 — Supplementary file1 (DOCX 197 kb) [file 455_2024_10673_MOESM1_ESM.docx]

# Appendix


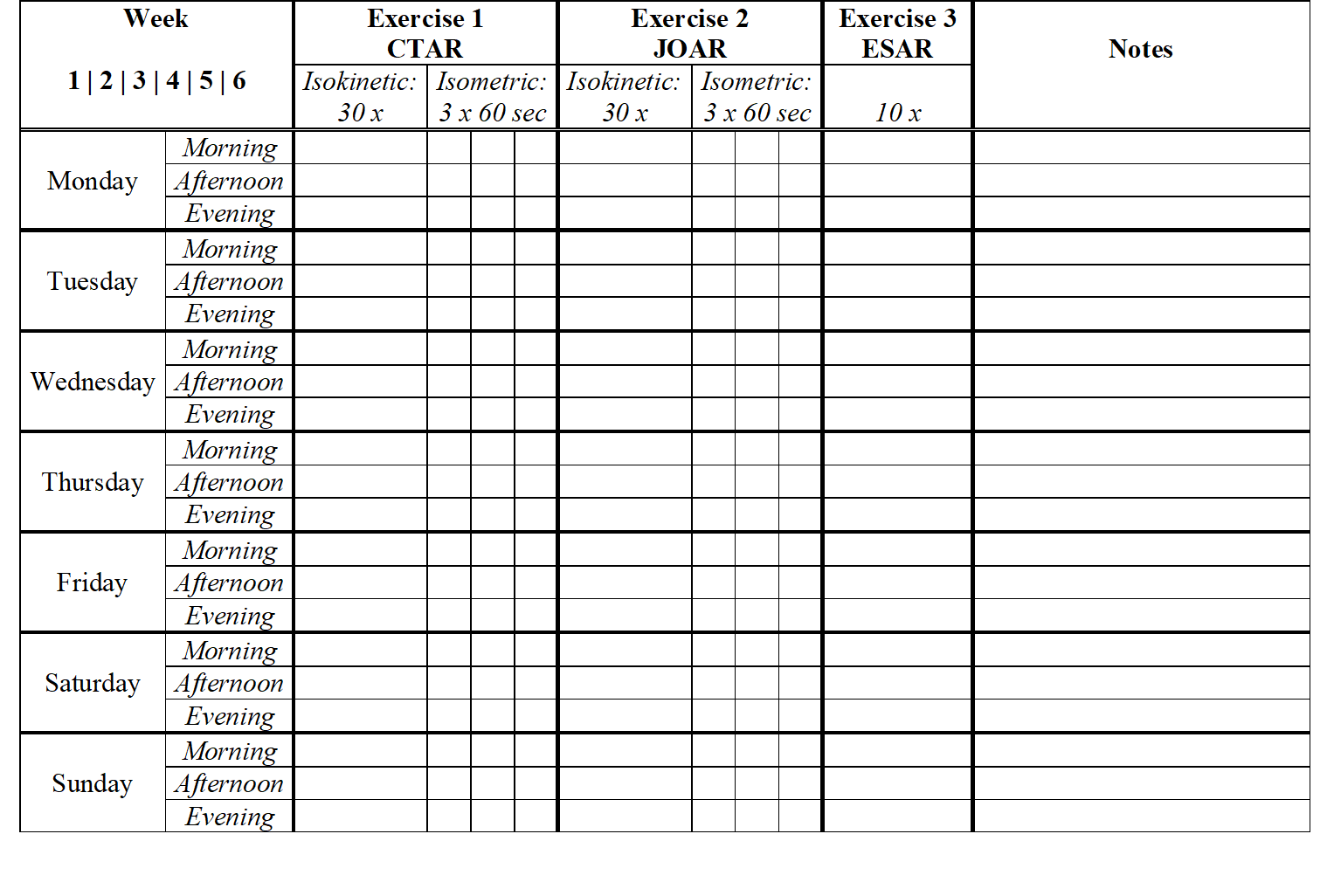


Figure 14 The study specific training logbook


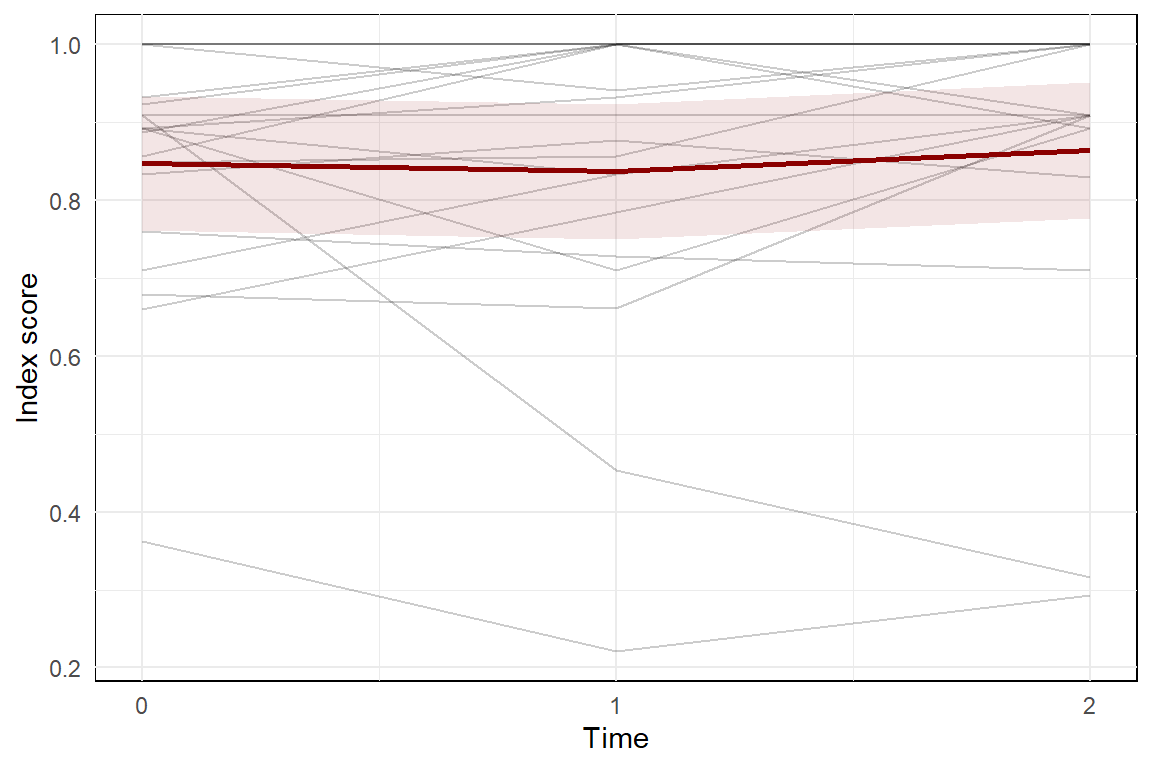


Figure 15 The validated EQ-5D-5L questionnaire focuses on mobility, self-care, usual activities, pain/discomfort, and anxiety/depression. The index outcome ranges from -.446 to 1.000, with a higher score indicating better health state. Each gray line represents one participant, while the red line represents the predicted marginal mean from the LME model, with the pink shading indicating the 95% confidence interval.


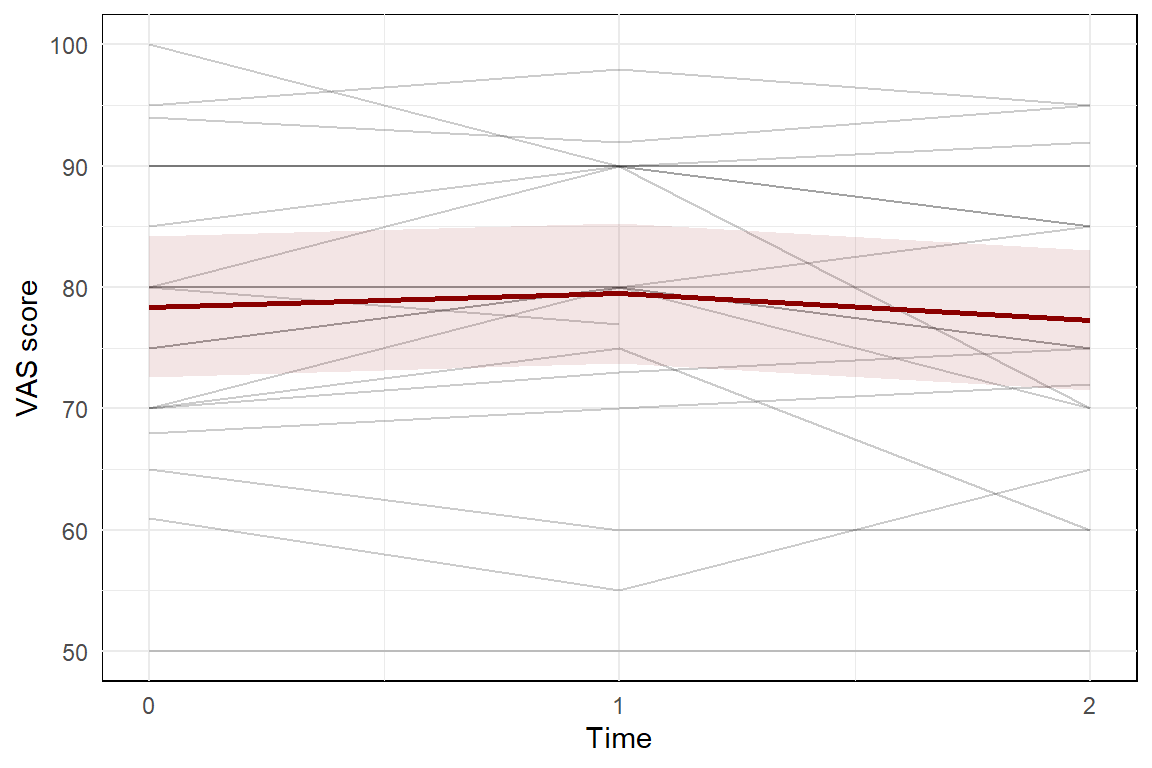


Figure 16 The outcomes per participant on the vertical visual analog scale (VAS) ranging from 0-100 to record the participants' self-rated health status. Each gray line represents one participant, while the red line represents the predicted marginal mean from the LME model, with the pink shading indicating the 95% confidence interval.


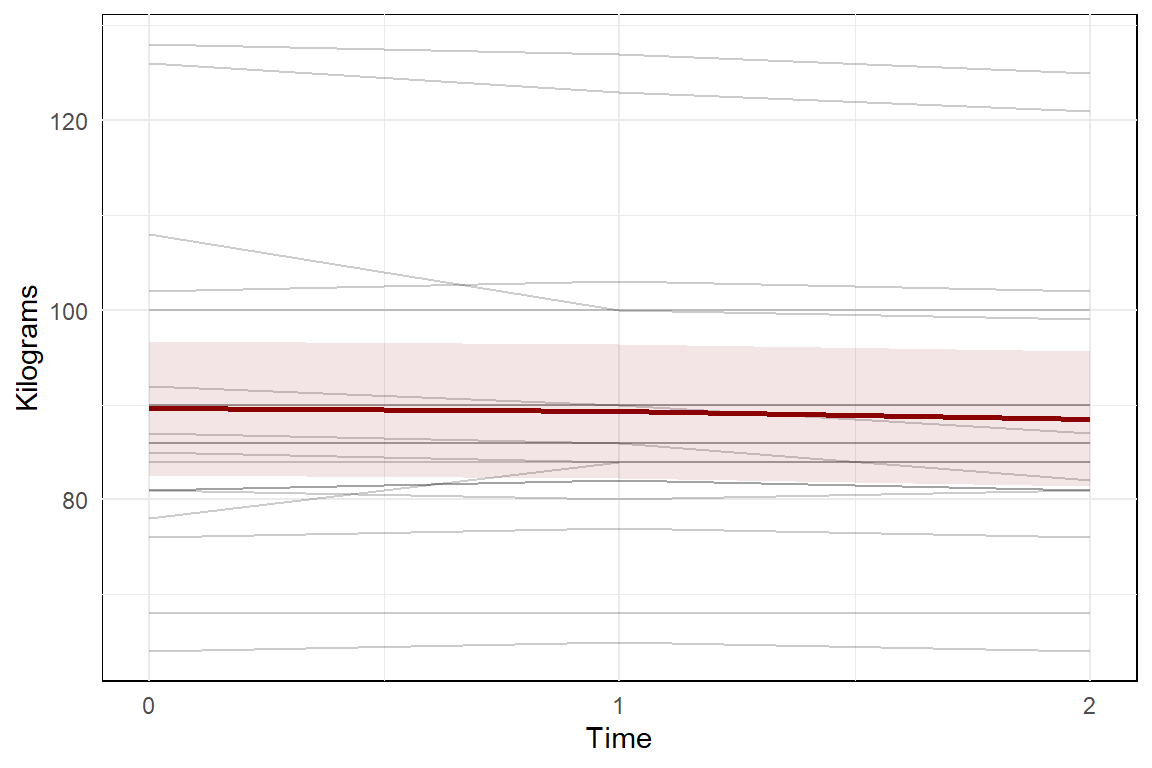


Figure 17 The bodyweight in kilograms per participant over time. Each gray line represents one participant, while the red line represents the predicted marginal mean from the LME model, with the pink shading indicating the 95% confidence interval.


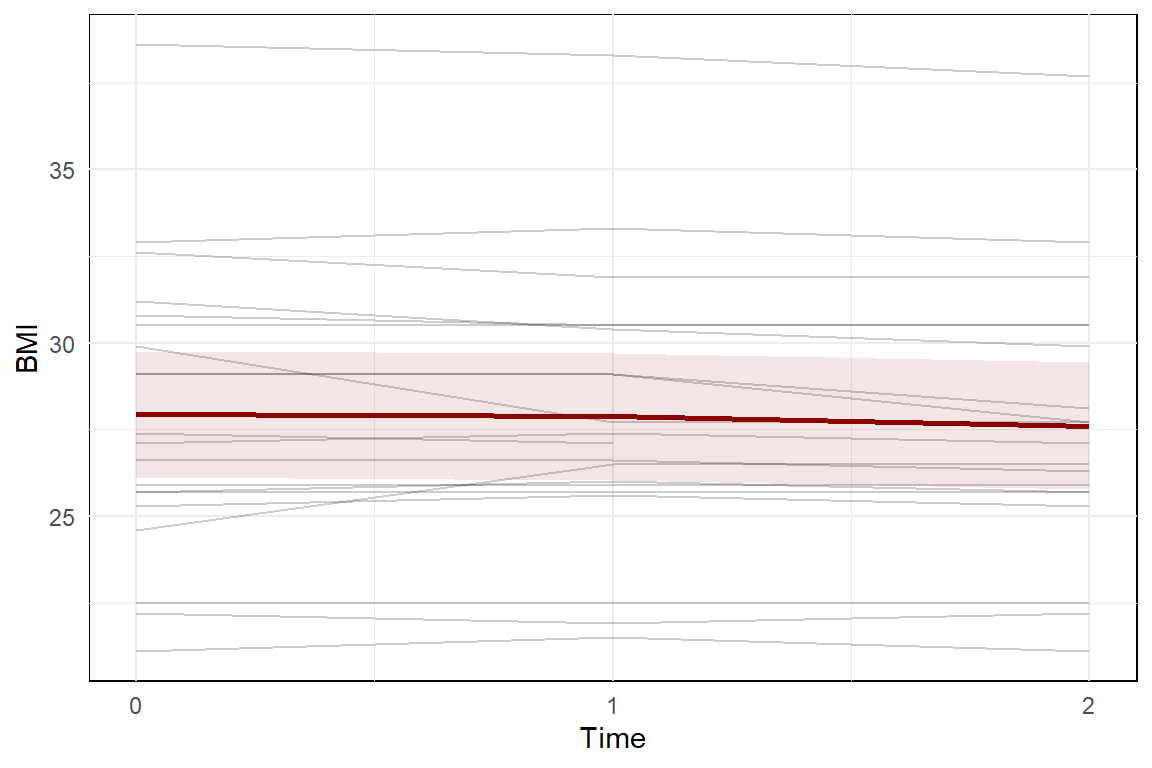


Figure 18 The Body Mass Index (BMI) scores per participant over time. Each gray line represents one participant, while the red line represents the predicted marginal mean from the LME model, with the pink shading indicating the 95% confidence interval.


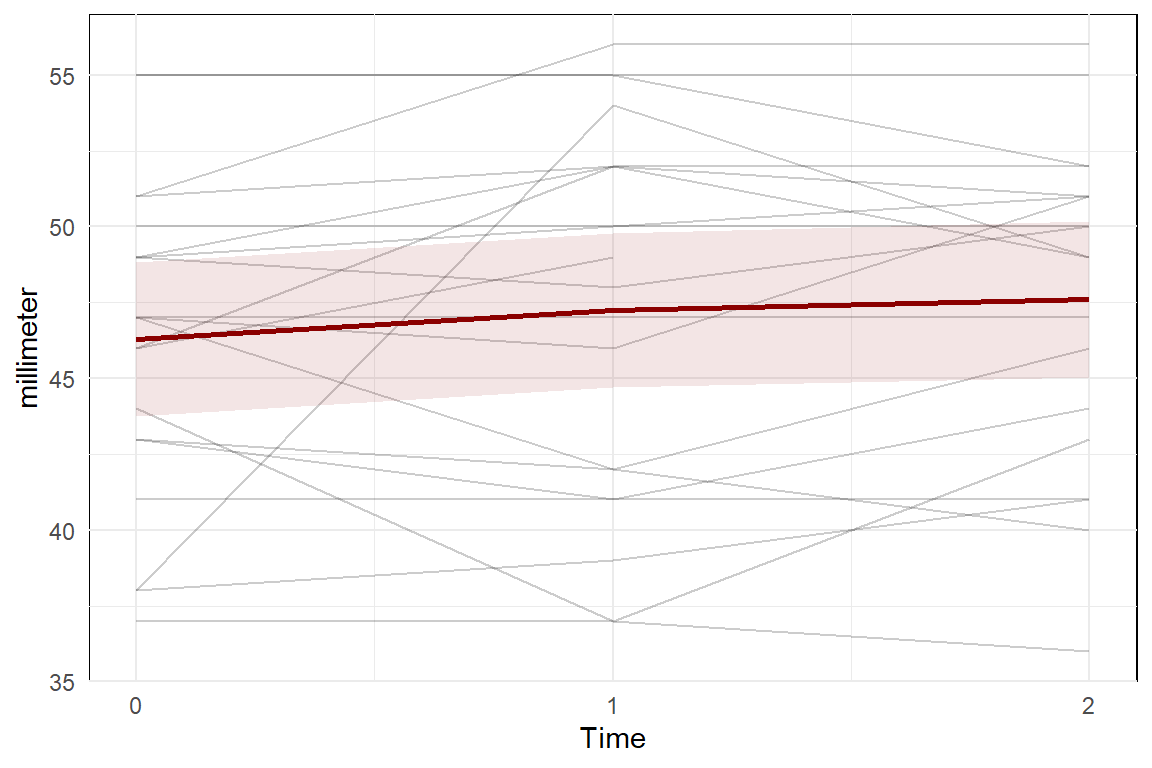


Figure 19 Maximum Interincisor Opening (MIO) of the mouth measured in millimeters using the TheraBite Range of Motion (ROM) Scale. Each gray line represents one participant, while the red line represents the predicted marginal mean from the LME model, with the pink shading indicating the 95% confidence interval.


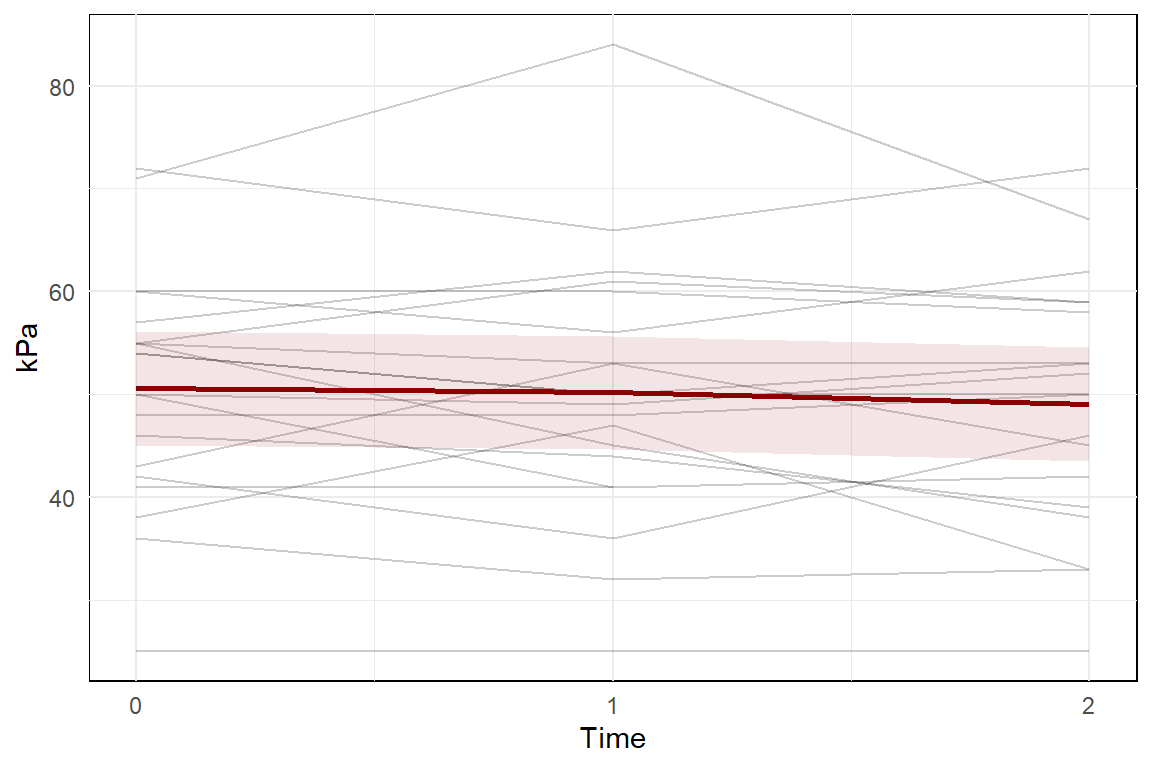


Figure 20 The Iowa Oral Performance Instrument (IOPI) anterior tongue strength in kPa per participant. Each gray line represents one participant, while the red line represents the predicted marginal mean from the LME model, with the pink shading indicating the 95% confidence interval.

Figure 21 Training level of the SEA 2.0 for the CTAR isokinetic exercise per participant per week

Figure 22 Training level of the SEA 2.0 for the CTAR isometric exercise per participant per week

Figure 23 Training level of the SEA 2.0 for the JOAR isokinetic exercise per participant per week

Figure 24 Training level of the SEA 2.0 for the JOAR isometric exercise per participant per week

Figure 25 Training level of the SEA 2.0 for the ESAR exercise per participant per week

*Questionnaire 1 Study specific questionnaire at T1*

| **Study specific questionnaire T1 SEATTLE \| N21STL Study**   \| Name: \| _____________ \| \| --- \| --- \| \| Date of birth: \| _____________ \|   ***Please fill in this questionnaire at the T1 assessment visit at the end of the six week training*** | |
| --- | --- | --- | --- | --- | --- |
| **1** | **Have you performed your exercises three times a day?**  1 = yes (continue to question 6)  2 = no, I have exercised approximately … times a **day**  3 = no, I have exercised approximately … times a **week** |
| **2** | **After how many days did you stop with your exercises?**  After ___ days |
| **3** | **Why did you stop with your exercises?** |
| **4** | **Did you re-continue your exercises after having stopped earlier?**  1 = yes  2 = no (continue to question 6) |
| **5** | **After how many days did you re-continue**  After ___ days |
| **6** | **How many days did you perform the exercises in total?**  In total ___ days |
| **7** | **How did you experience the exercises?**  1 = very unpleasant  2 = a bit unpleasant  3 = nor unpleasant or pleasant  4 = quite pleasant  5 = very pleasant |
| **8** | **Can you try to explain why?** |
| **9** | **How many days did it take to get used to the exercises?**  Approximately ___ days |
| **10** | **Did you have the feeling to benefit from the exercises?**  1 = not at all  2 = a little bit  3 = quite a bit  4 = very much |
| **11** | **After how many days, if any, did you notice the benefit(s)?**  Approximately ___ days |
| **12** | **Did you have problems getting used to or performing the exercises?** |
| **13** | **What is your general impression of the exercises?** |
| **14** | **Would you keep practicing, if recommended by your therapist?**  1 = yes  2 = probably  3 = probably not  4 = no |
| **15** | **Do you have (other) general remarks on the SEA 2.0?** |
| ***The following questions are focused on the assessments*** | |
| **16** | **What do you think of the number of assessments?**  1 = too few  2 = exactly right  3 = too much |
| **17** | **How did you experience the assessments?**  1 = very unpleasant  2 = a bit unpleasant  3 = nor unpleasant or pleasant  4 = quite pleasant  5 = very pleasant |
| **18** | **Can you try to explain why?** |
| **19** | **Do you have (other) general remarks on the assessments?** |

Questionnaire 2 Study specific questionnaire at T2

| **Study specific questionnaire T2 SEATTLE \| N21STL Study**   \| Name: \| _____________ \| \| --- \| --- \| \| Date of birth: \| _____________ \|   ***Please fill in this questionnaire at the T2 assessment visit at the end of the eight weeks rest period.*** | |
| --- | --- | --- | --- | --- | --- |
| **1** | **Have you performed your exercises during the eight week rest period?**  1 = yes (continue to question 2)  2 = no (continue to question 6) |
| **2** | **After how many days did you (re-)continue the exercises?**  After ___ days |
| **3** | **How many days did you perform the exercises in total?**  In total ___ days |
| **4** | On what training levels have you trained during the rest period?   \|  \| Min \| Max \| \| --- \| --- \| --- \| \| Level exercise 1 (CTAR) \|  \|  \| \| Level exercise 2 (JOAR) \|  \|  \| \| Level exercise 3 (ES) \|  \|  \| |
| **5** | **Can you try to explain why you (re-)continued the exercises?** |
| **6** | **Did you notice a difference or change in swallowing during the rest period?**  1 = yes (continue to question 7)  2 = no (continue to question 8) |
| **7** | **Can you try to explain the difference or changes?** |
| **8** | **Did you notice a difference or change in speech or voice during the rest period?**  1 = yes (continue to question 9)  2 = no (continue to question 10) |
| **9** | **Can you try to explain the difference or changes?** |

| **10** | **Did you notice a difference or change in other things during the rest period? (for instance: edema, neuralgia, muscle weakness)**  1 = yes (continue to question 11)  2 = no (continue to question 12) |
| --- | --- |
| **11** | **Can you try to explain the difference or changes?** |
| **12** | **Now that the rest period is over, do you intend to continue the SEA 2.0 exercises?**  1 = yes  2 = no |
| **13** | **Is there anything else I haven’t asked that I should know for the research?** |
